# Prescribing Patterns and Treatment Persistence for Overactive Bladder in Japan Across Medical Specialties and Facility Types: A Nationwide Claims Database Study

**DOI:** 10.1111/iju.70468

**Published:** 2026-04-21

**Authors:** Kenji Omae, Keita Hashimoto, Shotaro Maeda

**Affiliations:** ^1^ Department of Innovative Research and Education for Clinicians and Trainees (DiRECT), Fukushima Medical University Hospital Fukushima Japan; ^2^ Medical Affairs, Kyorin Pharmaceutical Co., Ltd. Tokyo Japan

**Keywords:** β3‐adrenoceptor agonists, anticholinergic agents, claims databases, overactive urinary bladder, treatment adherence and compliance

## Abstract

**Objectives:**

To describe real‐world initial pharmacotherapy, treatment persistence, and diagnostic testing for overactive bladder in Japan by medical specialty and facility type.

**Methods:**

This retrospective descriptive study used nationwide administrative claims data. Patients aged 15 years or older diagnosed with overactive bladder in 2022 who newly initiated pharmacotherapy were followed for 1 year. Analyses were conducted across four groups defined by medical specialty and facility type. We summarized initial medication, baseline comorbidities, one‐year treatment persistence, and performance of urinalysis and postvoid residual volume measurement at diagnosis and within 1–3 months thereafter.

**Results:**

Of 702 078 patients diagnosed with overactive bladder, 65 173 met the eligibility criteria (median age 80 years; interquartile range 74.0–85.0; women 56.3%). β3‐adrenoceptor agonists accounted for more than 70% of initial prescriptions in both urology and internal medicine, whereas anticholinergics were used more often in internal medicine than in urology. Baseline comorbidities, including dementia, were broadly similar between patients initiating β3‐adrenoceptor agonists and those initiating anticholinergics, except for benign prostatic hyperplasia. At 1 year, persistence was higher for β3‐adrenoceptor agonists than for anticholinergics and other drugs, but remained below 30% for β3‐adrenoceptor agonists. Urinalysis and postvoid residual volume measurement were performed more frequently in urology than in internal medicine.

**Conclusions:**

β3‐adrenoceptor agonists predominated as initial pharmacotherapy for overactive bladder in Japan, whereas anticholinergics were used more often in internal medicine than in urology. Similar comorbidity profiles and specialty‐related differences in diagnostic testing highlight opportunities to optimize individualized prescribing and strengthen guideline‐concordant evaluation across care settings.

## 1 Introduction

Overactive bladder (OAB) is defined by urinary urgency as the cardinal symptom, usually accompanied by increased daytime frequency and nocturia. In Japanese epidemiological studies, the prevalence of OAB in individuals aged ≥ 40 years is 13.8% and increases with age [1]; the mean age of patients receiving treatment is 74 years [2]. OAB and related symptoms such as nocturia are associated with sleep disturbance, depression, and increased risk of falls, impairing quality of life (QoL) and burdening healthcare and long‐term care systems [3, 4, 5].

Although lifestyle modification and behavioral therapy are also recommended, pharmacotherapy is the mainstay of management, with β3‐adrenoceptor agonists and anticholinergics being widely used [6]. Both drug classes have distinct adverse‐effect profiles. Anticholinergics may cause dry mouth, constipation, and cognitive impairment, whereas β3‐adrenoceptor agonists may induce tachycardia and increase blood pressure [7, 8]. These risks are particularly relevant in older adults, who commonly have multiple comorbidities, polypharmacy, and reduced organ function, necessitating careful drug selection. Thus, the choice of OAB medication varies based not only on the patient's symptoms but also on their comorbidities and concomitant medications.

Recent studies have focused on factors beyond patient characteristics that influence prescribing patterns for OAB treatment. A study using Medicare Part D claims data in the United States found substantial variations in prescribing practices for β3‐adrenoceptor agonists and anticholinergics by physician specialty and practice setting [9]. A Japanese study using medical claims data also reported differences in initial prescribing patterns of these medications by medical specialty and facility type. However, the number of patients aged ≥ 65 years included in that study was extremely limited [10].

Therefore, to better understand differences in OAB management practices in Japan by medical specialty and facility type, we expanded the study population to include older adults and analyzed a broader range of medications using a nationwide claims database. Furthermore, we examined various practice patterns, including not only initial drug selection but also treatment persistence rates and the implementation status of urinalysis and postvoid residual volume (PVR) measurements when initiating prescriptions. These assessments are recommended in the OAB clinical practice guidelines as part of the pretreatment work‐up to evaluate underlying conditions such as urinary tract infections and voiding dysfunction [6].

## 2 Methods

### 2.1 Data Source and Study Population

This retrospective descriptive study used the DeSC claims database (DeSC Healthcare Inc., Tokyo, Japan), which contains anonymized health insurance claims from public and employer‐based insurers in Japan [11]. The DeSC database comprises claims data from Japan's three major public health insurance systems: Society‐Managed Health Insurance (large company employees and dependents), National Health Insurance (self‐employed, farmers, students, and retirees under 75), and the Latter‐Stage Elderly Healthcare System (individuals aged ≥ 75 years). The database includes data for approximately 20 million beneficiaries, representing approximately 17% of Japan's total population. This study adhered to the Reporting of studies Conducted using Observational Routinely‐collected Data (RECORD) guidelines [12], and institutional review board approval and informed consent were waived because only anonymized data were used, in accordance with Japanese ethical guidelines [13].

**FIGURE 1:**
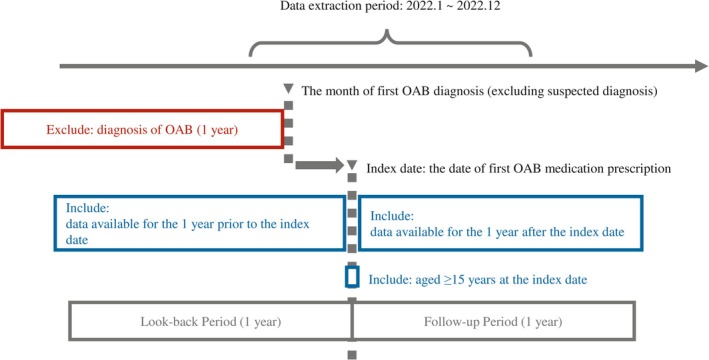
Patient selection algorithm. OAB, Overactive bladder.

Using Japan‐specific disease codes (8 844 583 and 8 843 031), we identified patients diagnosed with OAB in 2022, excluding those with only “suspected” OAB. Among these patients, we selected those who received a prescription for one of the OAB medications listed in the Japanese clinical practice guidelines [6] (Anatomical Therapeutic Chemical Classification System code G04D4 and V03B1 [Kampo medicine, ingredient name “Gosha‐jinki‐gan”]). We defined the date of the first prescription as the index date (full definitions are listed in Table S1). To construct a new‐user cohort, patients were required to have no prior diagnosis of OAB within 1 year before their OAB diagnosis. The 1‐year period before the index date was defined as the look‐back period to identify comorbidities and prior treatments, and the 1‐year period after the index date as the follow‐up period. To minimize information bias from incomplete observation, patients with data loss during either period were excluded. Patients aged < 15 years and those who had a record of OAB diagnoses from multiple departments during the index month were excluded. An overview of the patient selection algorithm is shown in Figure 1.

### 2.2 Study Variables

Age and sex at the index date; the medical specialty and size of the facility where OAB medications were first prescribed; and comorbidities recorded during the look‐back period were extracted as patient background characteristics. Facilities were classified into two types based on size: clinics (≤ 19 beds) and hospitals (≥ 20 beds). This classification is based on the Japanese Medical Care Act, under which facilities with 19 or fewer beds are legally defined as clinics, whereas those with 20 or more beds are classified as hospitals. Clinics typically function as primary care settings, while hospitals provide secondary or tertiary care and are more likely to have specialized departments and structured follow‐up systems. Regarding the initial OAB medication, patients who received ≥ 2 OAB medications concomitantly on the index date were classified as “Multiple.” Comorbidities included common conditions such as hypertension, diabetes, and dyslipidemia, as well as conditions linked to OAB medication‐related risks in older adults [1, 7, 14], including dementia, glaucoma, and cardiovascular disease (defined as heart failure, angina pectoris, myocardial infarction, atrial fibrillation/flutter, and arrhythmia). These comorbidities were defined using International Classification of Diseases, 10th Revision (ICD‐10) codes, excluding suspected diagnoses, consistent with previous studies. Sensitivity analyses evaluated these comorbidities based on different definitions combining relevant medication prescriptions and the ICD‐10 codes. Urinalysis and PVR measurement were defined by Japanese medical procedure codes, and we assessed whether they were performed at OAB diagnosis and within the subsequent 1–3 months. Detailed definitions are provided in Table S2.

Medication persistence was evaluated as the period from the index date until medication change or treatment discontinuation. Treatment discontinuation was defined as the absence of a subsequent prescription within 30 days after the theoretical end date of the preceding prescription.

### 2.3 Statistical Analyses

Patient background characteristics, prescribed medications, tests performed, and medication persistence were descriptively analyzed. We focused on the urology and internal medicine departments as these were the departments most frequently visited by patients with OAB (*n* = 53 433, 82.0%), and defined four groups based on medical specialty and facility type: Internal medicine, Clinic; Internal medicine, Hospital; Urology, Clinic; and Urology, Hospital. Using the same classification, we summarized comorbidities among patients who initiated pharmacotherapy with either drug class (β3‐adrenoceptor agonists or anticholinergics).

Continuous variables were presented as medians (interquartile range [IQR]), and categorical variables as numbers and percentages. Medication persistence was evaluated using Kaplan–Meier curves for each drug and therapeutic class. Patients were censored at the end of the 365‐day follow‐up period. Given the descriptive study design, no comparative statistical tests were performed. Analyses were conducted using the R software, version 4.5.2 (R Foundation for Statistical Computing, Vienna, Austria).

## 3 Results

### 3.1 Study Population

**FIGURE 2:**
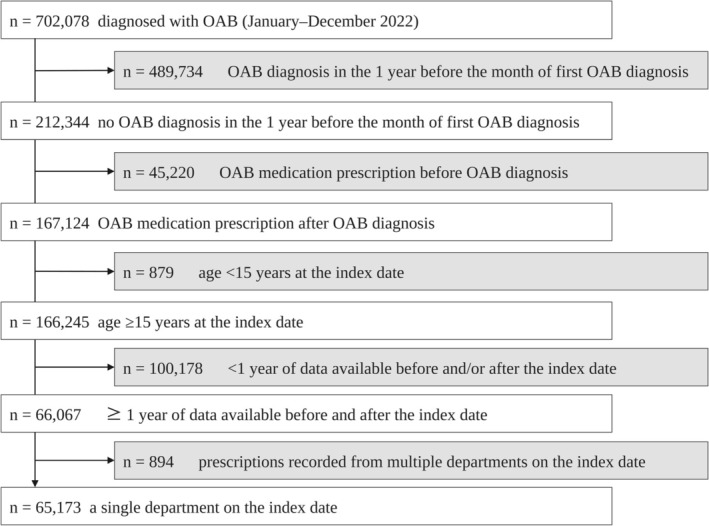
Overview of the enrolled patients. OAB, Overactive bladder.

A total of 702 078 patients diagnosed with OAB between January and December 2022 were identified. After applying inclusion and exclusion criteria, 65 173 patients were included in the analysis (Figure 2).

### 3.2 Baseline Characteristics

Among the 65 173 patients, the median age was 80 years (IQR, 74.0–85.0) and 56.3% were women. In urology departments, male patients accounted for a larger proportion at both clinics and hospitals, whereas in internal medicine departments, the proportion of women was higher. Among patients treated in urology departments, benign prostatic hyperplasia (BPH) was more prevalent than in internal medicine departments, with prevalence of 44.0% and 55.4% in urology clinics and hospitals, respectively, compared with 17.6% and 28.9% in internal medicine clinics and hospitals (Table 1). Data on comorbidities defined using combined diagnosis and medication codes in the sensitivity analysis are presented in Table S3. Under this definition, the prevalence of comorbidities was generally lower than that reported in Table 1, particularly for type 2 diabetes, heart failure, and arrhythmia.

**TABLE 1:** Patient Characteristics. Baseline characteristics of patients with OAB, stratified by medical specialty and facility type. Values are presented as median (IOR) or *n* (%), as appropriate. Percentages are calculated using the total number of patients in each column as the denominator. Comorbidities were identified during the 1‐year look‐back period using ICD‐10 diagnosis codes in claims data (see Table S2 for code definitions).

	Overall	Internal medicine, clinic	Internal medicine, hospital	Urology, clinic	Urology, hospital
	*n* = 65 173	*n* = 24 751	*n* = 5855	*n* = 15 142	*n* = 7685
Age					
Median (IQR)	80.0 (74.0–85.0)	81.0 (77.0–86.0)	82.0 (78.0–86.0)	78.0 (69.0–83.0)	80.0 (74.0–84.0)
Gender, *n* (%)					
Female	36 669 (56.3%)	16 258 (65.7%)	3234 (55.2%)	7228 (47.7%)	2337 (30.4%)
Insurance type, *n* (%)					
Health insurance society	6307 (9.7%)	1730 (7.0%)	291 (5.0%)	2692 (17.8%)	654 (8.5%)
Medical Care System for the Elderly	48 693 (74.7%)	19 458 (78.6%)	4832 (82.5%)	9653 (63.7%)	5701 (74.2%)
National health insurance	10 173 (15.6%)	3563 (14.4%)	732 (12.5%)	2797 (18.5%)	1330 (17.3%)
Comorbidity, *n* (%)					
Hypertension	45 813 (70.3%)	18 677 (75.5%)	4513 (77.1%)	9050 (59.8%)	5188 (67.5%)
Type 2 diabetes	24 545 (37.7%)	9537 (38.5%)	2633 (45.0%)	5107 (33.7%)	3049 (39.7%)
Dyslipidemia	36 815 (56.5%)	14 904 (60.2%)	3410 (58.2%)	7758 (51.2%)	4079 (53.1%)
Dementia	6596 (10.1%)	2790 (11.3%)	865 (14.8%)	958 (6.3%)	485 (6.3%)
Glaucoma	8928 (13.7%)	3407 (13.8%)	815 (13.9%)	2019 (13.3%)	1097 (14.3%)
Dry mouth/Xerostomia	939 (1.4%)	349 (1.4%)	90 (1.5%)	197 (1.3%)	95 (1.2%)
Constipation	30 066 (46.1%)	11 278 (45.6%)	3362 (57.4%)	5857 (38.7%)	3589 (46.7%)
Benign prostatic hyperplasia	19 298 (29.6%)	4349 (17.6%)	1694 (28.9%)	6660 (44.0%)	4256 (55.4%)
Cerebrovascular diseases (Stroke)	13 023 (20.0%)	4683 (18.9%)	1396 (23.8%)	2704 (17.9%)	1485 (19.3%)
Myocardial infarction	916 (1.4%)	318 (1.3%)	92 (1.6%)	191 (1.3%)	146 (1.9%)
Heart failure	16 515 (25.3%)	6542 (26.4%)	1962 (33.5%)	3008 (19.9%)	1875 (24.4%)
Angina pectoris	11 916 (18.3%)	4414 (17.8%)	1338 (22.9%)	2372 (15.7%)	1553 (20.2%)
Atrial fibrillation/Flutter	5611 (8.6%)	1959 (7.9%)	607 (10.4%)	1208 (8.0%)	701 (9.1%)
Arrhythmia	8741 (13.4%)	3390 (13.7%)	777 (13.3%)	1908 (12.6%)	1048 (13.6%)

Abbreviations: ICD‐10, International Statistical Classification of Diseases and Related Health Problems 10th Revision; IQR, interquartile range; OAB, overactive bladder.

### 3.3 Initial Pharmacological Treatment for OAB

**FIGURE 3:**
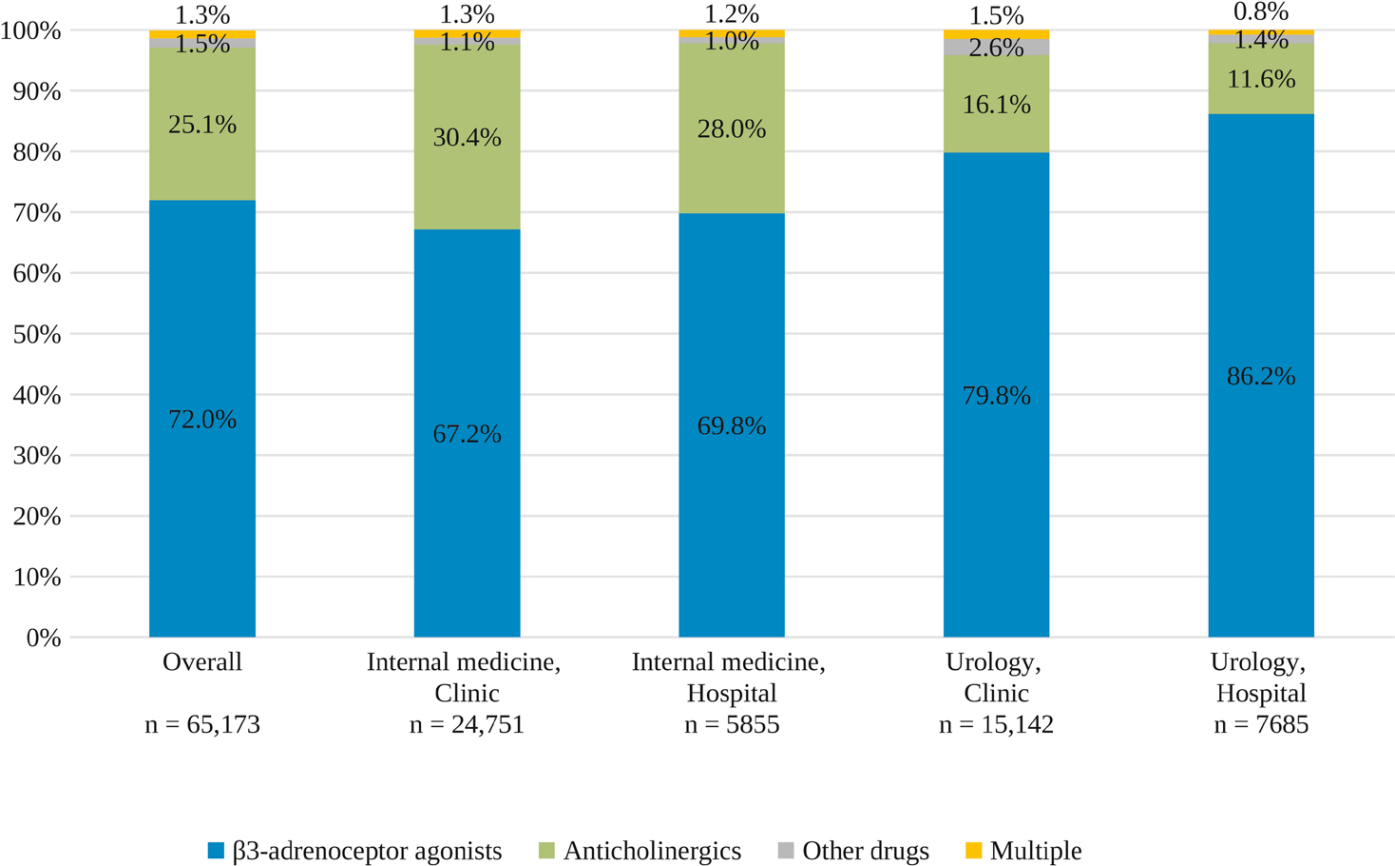
Proportions of initial pharmacotherapy of OAB by medical specialty and facility type. Bars represent the percentage of patients who initiated treatment with β3‐adrenoceptor agonists, anticholinergics, other drugs, and multiple OAB medications. Multiple indicates concomitant prescriptions of ≥ 2 OAB medications on the index date. Anticholinergics include fesoterodine, imidafenacin, oxybutynin (transdermal and oral formulations), propiverine, solifenacin, and tolterodine. β3‐adrenoceptor agonists include mirabegron and vibegron. Others include flavoxate and the Kampo formulation Gosha‐jinki‐gan. *n* indicates the number of patients in each subgroup. OAB, Overactive bladder.

In both urology and internal medicine departments, β3‐adrenoceptor agonists were the most frequently prescribed initial OAB medications in both men and women (Figure 3; Table S4). While anticholinergics accounted for 11.6% and 16.1% of initial prescriptions in urology departments in hospitals and clinics, respectively, they accounted for 28.0% and 30.4% in internal medicine departments. The proportion of patients initiating β3‐adrenoceptor agonists was generally higher in men than in women across specialty–facility strata (Table S4). The proportions of individual OAB medications varied by patient sex and facility type (Table S5).

### 3.4 Prevalence of Comorbidities by Initial OAB Medication

The distribution of comorbidities by initial OAB medication is summarized in Table 2. The prevalence of comorbidities was broadly similar between patients receiving β3‐adrenoceptor agonists and those receiving anticholinergics across medical specialties and facility types, except for BPH (Table 2). Among patients who initially received anticholinergics, 11.7% had comorbid dementia. Drug‐specific results are presented in Table S6. Among patients with BPH, the proportion receiving concomitant BPH medications is summarized in Table S7.

**TABLE 2:** Baseline comorbidities by initial OAB medication. Baseline comorbidities of patients initiating pharmacotherapy for OAB, stratified by initial drug class (β3‐adrenoceptor agonists vs. anticholinergics), medical specialty, and facility type. Initial treatment was restricted to monotherapy (no concomitant OAB medications on the index date). Values are presented as *n* (%), where percentages are calculated using the total number of patients in each column (shown in the column headers) as the denominator. Comorbidities were identified during the 1‐year look‐back period using diagnosis codes in claims data (see Table S2 for code definitions).

Comorbidity, *n* (%)	Overall		Internal medicine, clinic		Internal medicine, hospital		Urology, clinic		Urology, hospital	
	β3‐adrenoceptor agonists	Anticholinergics	β3‐adrenoceptor agonists	Anticholinergics	β3‐adrenoceptor agonists	Anticholinergics	β3‐adrenoceptor agonists	Anticholinergics	β3‐adrenoceptor agonists	Anticholinergics
	(*n* = 46 918)	(*n* = 16 388)	(*n* = 16 624)	(*n* = 7519)	(*n* = 4088)	(*n* = 1638)	(*n* = 12 079)	(*n* = 2441)	(*n* = 6626)	(*n* = 891)
Hypertension	32 886 (70.1%)	11 642 (71.0%)	12 538 (75.4%)	5693 (75.7%)	3160 (77.3%)	1257 (76.7%)	7301 (60.4%)	1364 (55.9%)	4499 (67.9%)	576 (64.6%)
Type 2 diabetes	18 029 (38.4%)	5829 (35.6%)	6537 (39.3%)	2767 (36.8%)	1909 (46.7%)	670 (40.9%)	4149 (34.3%)	764 (31.3%)	2657 (40.1%)	325 (36.5%)
Dyslipidemia	26 487 (56.5%)	9263 (56.5%)	10 006 (60.2%)	4548 (60.5%)	2407 (58.9%)	925 (56.5%)	6238 (51.6%)	1183 (48.5%)	3520 (53.1%)	460 (51.6%)
Dementia	4471 (9.5%)	1921 (11.7%)	1814 (10.9%)	890 (11.8%)	532 (13.0%)	308 (18.8%)	754 (6.2%)	165 (6.8%)	435 (6.6%)	40 (4.5%)
Glaucoma	6667 (14.2%)	1993 (12.2%)	2349 (14.1%)	955 (12.7%)	603 (14.8%)	190 (11.6%)	1704 (14.1%)	249 (10.2%)	956 (14.4%)	116 (13.0%)
Dry mouth/Xerostomia	677 (1.4%)	232 (1.4%)	242 (1.5%)	98 (1.3%)	72 (1.8%)	18 (1.1%)	161 (1.3%)	27 (1.1%)	77 (1.2%)	16 (1.8%)
Constipation	21 493 (45.8%)	7663 (46.8%)	7536 (45.3%)	3437 (45.7%)	2281 (55.8%)	1003 (61.2%)	4732 (39.2%)	872 (35.7%)	3107 (46.9%)	401 (45.0%)
Benign prostatic hyperplasia	15 839 (33.8%)	3014 (18.4%)	3295 (19.8%)	942 (12.5%)	1382 (33.8%)	285 (17.4%)	5608 (46.4%)	876 (35.9%)	3760 (56.7%)	430 (48.3%)
Cerebrovascular diseases (Stroke)	9481 (20.2%)	3175 (19.4%)	3197 (19.2%)	1370 (18.2%)	965 (23.6%)	399 (24.4%)	2212 (18.3%)	381 (15.6%)	1294 (19.5%)	159 (17.8%)
Myocardial infarction	665 (1.4%)	225 (1.4%)	211 (1.3%)	102 (1.4%)	58 (1.4%)	30 (1.8%)	156 (1.3%)	26 (1.1%)	131 (2.0%)	13 (1.5%)
Heart failure	11 819 (25.2%)	4197 (25.6%)	4411 (26.5%)	1943 (25.8%)	1360 (33.3%)	557 (34.0%)	2398 (19.9%)	485 (19.9%)	1578 (23.8%)	243 (27.3%)
Angina pectoris	8648 (18.4%)	2910 (17.8%)	2991 (18.0%)	1306 (17.4%)	953 (23.3%)	355 (21.7%)	1884 (15.6%)	378 (15.5%)	1321 (19.9%)	190 (21.3%)
Atrial fibrillation/Flutter	4026 (8.6%)	1427 (8.7%)	1326 (8.0%)	583 (7.8%)	405 (9.9%)	185 (11.3%)	953 (7.9%)	212 (8.7%)	576 (8.7%)	105 (11.8%)
Arrhythmia	6286 (13.4%)	2178 (13.3%)	2287 (13.8%)	1007 (13.4%)	529 (12.9%)	220 (13.4%)	1491 (12.3%)	329 (13.5%)	894 (13.5%)	133 (14.9%)

Abbreviation: OAB, overactive bladder.

### 3.5 Treatment Persistence With Initial OAB Medications

**FIGURE 4:**
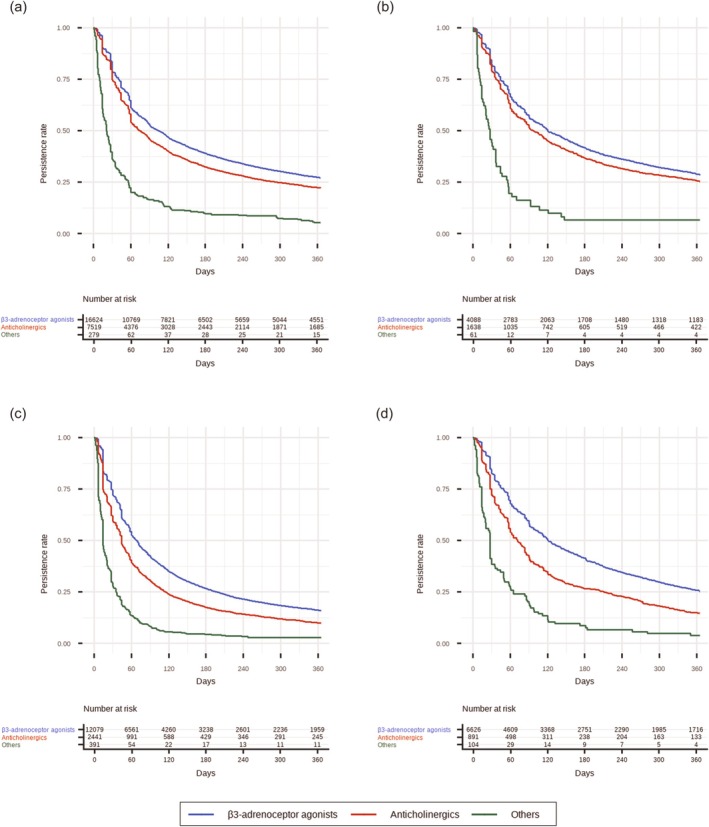
One‐year treatment persistence with initial OAB medications by drug class. (a) shows 1‐year treatment persistence in internal medicine clinics, (b) in internal medicine hospitals, (c) in urology clinics, and (d) in urology hospitals. Initial treatment was restricted to monotherapy (no concomitant OAB medications on the index date). Anticholinergics include fesoterodine, imidafenacin, oxybutynin (transdermal and oral formulations), propiverine, solifenacin, and tolterodine. β3‐adrenoceptor agonists include mirabegron and vibegron. Others include flavoxate and the Kampo formulation Gosha‐jinki‐gan. Abbreviation: OAB, overactive bladder.

At 1 year after initiation, treatment persistence was higher for β3‐adrenoceptor agonists than for anticholinergics and other drugs across all medical specialties and facility types; however, even the 1‐year persistence rate for β3‐adrenoceptor agonists remained below 30% (Figure 4). Persistence patterns were broadly similar across the four specialty–facility groups (Figure 4; Table S8), although persistence tended to be higher in internal medicine than in urology and higher in hospitals than in clinics. Figure S1 shows the differences in 1‐year persistence rates by medical specialty and facility type for individual medications. Drug‐specific persistence rates are provided in Table S8, and the median treatment durations are summarized in Table S9.

### 3.6 Urinalysis and PVR Measurement

**FIGURE 5:**
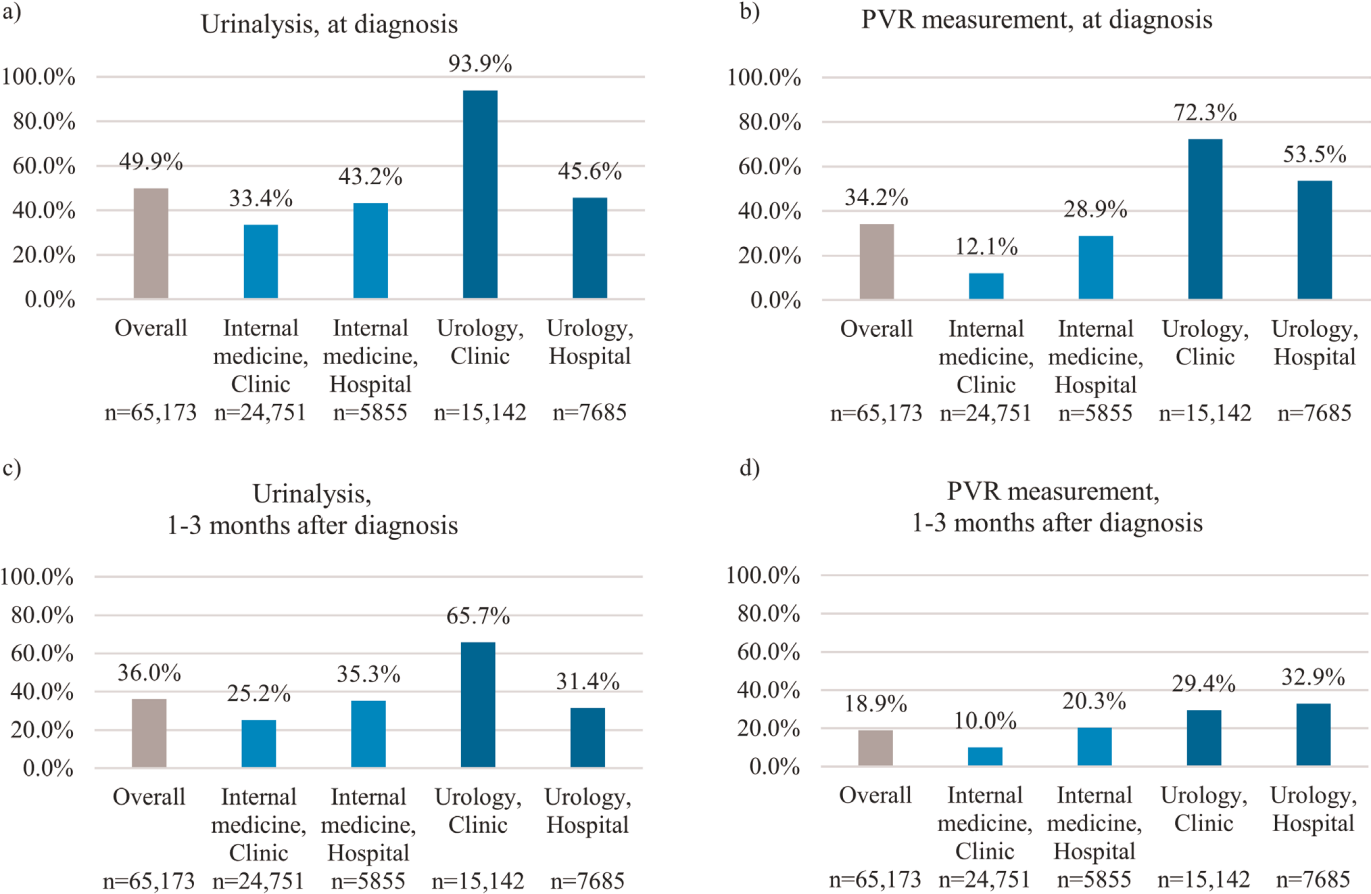
Proportions of patients undergoing urinalysis and PVR measurement at the time of overactive bladder diagnosis and during the subsequent 1–3 months, stratified by medical specialty and facility type. Panel (a) shows urinalysis at diagnosis; panel (b) shows PVR measurement at diagnosis; panel (c) shows urinalysis within 1–3 months after diagnosis; and panel (d) shows PVR measurement within 1–3 months after diagnosis. Percentages were calculated using the total number of patients in each subgroup (shown below each bar) as the denominator. Tests were identified using Japanese medical procedure/billing codes in claims data. PVR, postvoid residual volume.

The implementation rates of urinalysis and PVR measurement at diagnosis and 1–3 months thereafter differed markedly among the four groups. At diagnosis, both tests were conducted more frequently in urology than in internal medicine. Overall, urology clinics demonstrated the highest testing rates, except for PVR measurement at 1–3 months post‐diagnosis, while internal medicine clinics had the lowest rates at both diagnosis and 1–3 months post‐diagnosis (Figure 5).

## 4 Discussion

This study used a nationwide claims database to describe patterns of initial OAB medication selection, treatment persistence, and implementation of diagnostic tests according to medical specialty and facility type among patients aged ≥ 15 years with OAB in Japan. In urology departments, men comprised a larger proportion of patients than women, likely because men with BPH are more likely to receive care from urologists, whereas women may receive OAB prescriptions alongside medications for comorbidities in internal medicine departments. These findings suggest that treatment‐seeking behavior and healthcare utilization patterns vary by sex and comorbidity.

In both urology and internal medicine departments, β3‐adrenoceptor agonists were the predominant initial OAB medications, accounting for more than 70% of initial prescriptions, although anticholinergics were prescribed more often in internal medicine departments than in urology departments. This preference likely reflects their more favorable adverse‐event profile, including lower rates of dry mouth and constipation than anticholinergics [15]. Accumulating evidence regarding the impact of anticholinergic burden on cognitive function, particularly in older adults [14, 16], may further encourage clinicians to select β3‐adrenoceptor agonists as first‐line pharmacotherapy. In addition, across all specialty–facility strata, β3‐adrenoceptor agonists were prescribed more frequently in men than in women, especially in men with BPH. These patterns likely reflect concern about urinary retention due to bladder outlet obstruction with BPH and the perception that β3‐adrenoceptor agonists have a lower risk of urinary retention than anticholinergics [15]. Nevertheless, they are not free from this risk, and careful monitoring, including ultrasonographic assessment of PVR, remains important.

Comorbidities relevant to drug safety also warrant careful consideration, particularly in older adults where polypharmacy is common. In this study, 11.7% of patients who initially received anticholinergics had preexisting dementia identified during the 1‐year look‐back period before OAB treatment initiation; this does not imply that dementia was induced or worsened by anticholinergic therapy. Given the potential for anticholinergics to exacerbate cognitive impairment, more cautious use in patients with dementia would be desirable, and β3‐adrenoceptor agonists may offer a safer alternative. On the other hand, the Guidelines for medical treatment and its safety in the elderly list tachycardia and hypertension as precautions when prescribing β3‐adrenoceptor agonists [17]. Although the incidence of cardiovascular adverse events was only 0.48% for mirabegron [18] and 0.22% for vibegron [19] in early post‐marketing surveillance of β3‐adrenoceptor agonists, particular caution is advised in patients with cardiovascular disease. However, this study found that β3‐adrenoceptor agonists were prescribed even to patients with cardiovascular disease, and their prescribing patterns were similar to those of anticholinergics. Taken together, these findings underscore the need for careful, individualized prescribing for OAB that balances the distinct risk–benefit profiles of anticholinergics and β3‐adrenoceptor agonists, especially in older adults.

Beyond initial prescribing, we observed low treatment persistence in routine practice. In our cohort, one‐year treatment persistence with OAB medications was below 30% for all drug classes across all specialty‐facility groups, indicating that long‐term pharmacotherapy is difficult to maintain. These findings are consistent with previous reports [20], in which insufficient symptom improvement and adverse events were major reasons for discontinuation [21, 22]. Prior studies have also described a mismatch between patient expectations and physician treatment goals: patients often expect near‐complete symptom control, whereas physicians adopt more modest targets [23]. These observations highlight the importance of sharing treatment goals with patients and regularly assessing effectiveness, safety, and satisfaction. In this study, persistence tended to be higher in internal medicine than in urology and higher in hospitals than in clinics. Any specialty‐related differences in persistence may reflect differences in follow‐up patterns, with internal medicine more likely to continue regular visits for management of comorbid conditions such as hypertension and diabetes. Higher persistence in hospitals may likewise reflect more structured follow‐up and ongoing management of comorbidities. Notably, low persistence is not necessarily synonymous with inappropriate care. Some patients may discontinue therapy after adequate symptom improvement, switch to other OAB medications, or transition to alternative treatments such as surgery. Because reasons for discontinuation were unavailable, we could not judge appropriateness based solely on persistence data. Nevertheless, the challenges described above may contribute to difficulties in sustaining long‐term pharmacotherapy.

Implementation of urinalysis and PVR measurement at the time of diagnosis was more frequent in urology than in internal medicine. The OAB clinical practice guidelines recommend these tests before treatment, as part of the diagnostic algorithm, to detect hematuria, pyuria, and voiding dysfunction [6]. Appropriate implementation of these tests enables differential diagnosis between OAB and other conditions with similar symptoms, such as bladder cancers, urinary tract infections, and urinary stones. Furthermore, by performing appropriate evaluations in patients with voiding dysfunction or substantial PVR, the risk of adverse events such as urinary retention associated with the prescription of OAB medications can be reduced. To ensure the quality and safety of OAB management, it is necessary to disseminate the diagnostic algorithm more widely, not only among urologists but also among other specialties, including internal medicine, and to create an environment that facilitates implementation of recommended tests.

## 5 Strengths and Limitations

This study has several strengths. Unlike previous Japanese database studies of OAB that primarily used employer‐based insurance data and therefore included relatively younger, actively employed populations, we used a large database encompassing Society‐managed Health Insurance, National Health Insurance and the Latter‐Stage Elderly Healthcare System. Consequently, the study population included a substantial proportion of older adults (median age 80.0 years), allowing our findings to better reflect real‐world practice in older adults with OAB. The age distribution and sex ratio were generally consistent with a previous Japanese real‐world survey of patients receiving OAB medications [2], suggesting that our population reasonably reflects patients with OAB in Japan. In addition, we evaluated not only initial medication choices by medical specialty and facility type but also comorbidities, treatment persistence, and implementation of diagnostic tests, providing a multifaceted picture of pharmacological management in this population.

This study also has limitations. First, claims data do not include detailed clinical information such as OAB symptom severity, QoL, or patient preferences, and misclassification of OAB and comorbidity codes cannot be excluded. Implementation of urinalysis and PVR measurements was identified using billing codes; in Japan, diagnostic tests are generally covered by the national health insurance system and submitted as insurance claims, although tests that were performed but not billed may not have been captured. Second, medical specialty and facility type were defined only at the time of the initial prescription; subsequent care at referral facilities was not assessed. Third, treatment persistence was defined based on prescription records, and actual adherence was unknown. The observation period was limited to 1 year, although > 70% of medications were switched or discontinued within 1 year. These limitations should be considered when interpreting the findings. Fourth, the overall anticholinergic burden from concomitant medications other than OAB drugs was not assessed. As many older adults with OAB receive multiple medications with anticholinergic properties, the cumulative anticholinergic load may be higher than estimated from OAB prescriptions alone, which should be considered when interpreting the safety implications of our findings. Fifth, under certain bundled outpatient payment schemes in Japan, procedure codes for urinalysis, ultrasonography, and PVR measurement may not be individually recorded in claims data, potentially leading to underestimation of test implementation rates, particularly in non‐urology settings.

In conclusion, in this nationwide claims‐based study of 65 173 patients with OAB in Japan, β3‐adrenoceptor agonists were the predominant initial pharmacotherapy across urology and internal medicine, whereas anticholinergics were used more often in internal medicine. Baseline comorbidities, including dementia, were broadly similar between patients initiating β3‐adrenoceptor agonists and those initiating anticholinergics, except for BPH. Implementation of urinalysis and PVR measurement differed markedly by medical specialty and facility type, with higher testing rates in urology than in internal medicine. These findings highlight opportunities to optimize individualized prescribing and to strengthen guideline‐concordant diagnostic evaluation across care settings.

## Author Contributions

**Kenji Omae:** conceptualization; methodology; supervision; writing – review and editing. **Keita Hashimoto:** conceptualization; methodology; project administration; visualization; writing – original draft preparation; writing – review and editing. **Shotaro Maeda:** conceptualization; data curation; formal analysis; funding acquisition; investigation; methodology; resources; software; supervision; writing – original draft preparation; writing – review and editing.

## Funding

This work was supported by Kyorin Pharmaceutical.

## Consent

The authors have nothing to report.

## Conflicts of Interest

This work was funded by Kyorin. K.O. has received consultant fees from Kyorin. In addition, K.O. reports consultant fees and lecture fees from Astellas Pharma, and lecture fees from Pfizer, Nippon Shinyaku, and Kissei Pharmaceutical outside the submitted work. K.H. and S.M. are full‐time employees of Kyorin.

## Supporting information


**Figure S1:** One‐year treatment persistence for individual initial OAB medications. (a) shows 1‐year treatment persistence in internal medicine clinics, (b) in internal medicine hospitals, (c) in urology clinics, and (d) in urology hospitals. Initial treatment was restricted to monotherapy (no concomitant OAB medications on the index date). Abbreviations: OAB, overactive bladder; td, transdermal; po, per os.

**Table S1:** Definition of OAB diagnosis and OAB medications. Abbreviations: OAB, Overactive bladder; ATC, Anatomical Therapeutic Chemical Classification System.
**Table S2:** Definition of Comorbidities and tests.
**Table S3:** List of comorbidities defined by ICD‐10 diagnosis codes and ATC codes. Baseline comorbidities by medical specialty and facility type. Values are presented as *n* (%), with percentages calculated using the total number of patients in each column as the denominator. Comorbidities were defined using a claims‐based algorithm requiring both an ICD‐10 diagnosis code and a corresponding medication record during the 1‐year look‐back period (see Table S2 for code definitions). Dry mouth/xerostomia was not assessed using this combined diagnosis–medication definition because no established definition based on both diagnosis and medication codes has been reported in prior studies; therefore, results are shown as N/A.
**Table S4:** Initial prescription of OAB medications by medical specialty and facility type, stratified by sex. Initial pharmacotherapy for OAB by medical specialty, facility type, and sex. Values are presented as *n* (%). β3‐adrenoceptor agonists, anticholinergics, and other drugs were defined as described in Table S1. Multiple indicates concomitant prescriptions of ≥ 2 OAB medications on the index date. Abbreviations: OAB, overactive bladder.
**Table S5:** Proportion of initial OAB medications by drug across medical specialties, facility types, and sex. Values are presented as *n* (%) and represent the distribution of initial OAB pharmacotherapy by drug, stratified by medical specialty, facility type, and sex. Percentages are calculated using the total number of patients in each column as the denominator. Multiple indicates concomitant prescriptions of ≥ 2 OAB medications on the index date. Abbreviations: OAB, overactive bladder; td, transdermal; po, per os.
**Table S6:** Baseline comorbidities by initial OAB medication, stratified by medical specialty and facility type. Baseline comorbidities stratified by initial OAB medication and care setting. Initial treatment was restricted to monotherapy (no concomitant OAB medications on the index date). Values are presented as *n* (%), with percentages calculated using the total number of patients who initiated each medication (shown in the column headers) as the denominator. Results are shown for the overall cohort and separately for four specialty–facility groups (Internal medicine, Clinic; Internal medicine, Hospital; Urology, Clinic; and Urology, Hospital). Comorbidities were identified during the 1‐year look‐back period using ICD‐10 diagnosis codes in claims data (see Table S2 for code definitions). Cells are shown as N/A when no patients initiated the medication in that subgroup. Abbreviations: OAB, overactive bladder.
**Table S7:** Concomitant BPH medications among patients with BPH, stratified by medical specialty and facility type.
**Table S8:** Persistence rates of initial OAB medications at Days 90, 180, and 365, by medical specialty and facility type. Persistence was estimated using the Kaplan–Meier method. Values are treatment persistence estimates (%). *n* indicates the number of patients at baseline; *n* in parentheses indicates the number at risk at each time point. N/A, not estimable because no patients were at risk at the time point. Abbreviations: OAB, overactive bladder; td, transdermal; po, per os.
**Table S9:** Treatment duration of initial OAB medications (median [IQR]) by individual drug, medical specialty, and facility type. Treatment duration was defined as the number of days from the index date to medication change or treatment discontinuation, where discontinuation was defined as no subsequent prescription within 30 days after the end of the previous prescription. Patients who did not experience medication change or treatment discontinuation during follow‐up were censored at 365 days. Values are shown as median (IQR). *N* indicates the number of patients at baseline. N/A indicates not estimable due to no patients. Abbreviations: OAB, overactive bladder; td, transdermal; po, per os.

## Data Availability

Research data are not shared.
