# Supplementary material for: Prescribing Patterns and Treatment Persistence for Overactive Bladder in Japan Across Medical Specialties and Facility Types: A Nationwide Claims Database Study
Source: Int J Urol. 2026 Apr 21;33:e70468. doi: 10.1111/iju.70468 (PMC13098471; doi:10.1111/iju.70468)

Supplementary Figure S1. One-year treatment persistence for individual initial OAB medications. (a) shows 1-year treatment persistence in internal medicine clinics, (b) in internal medicine hospitals, (c) in urology clinics, and (d) in urology hospitals. Initial treatment was restricted to monotherapy (no concomitant OAB medications on the index date). Abbreviations: OAB, overactive bladder; td, transdermal; po, per os.


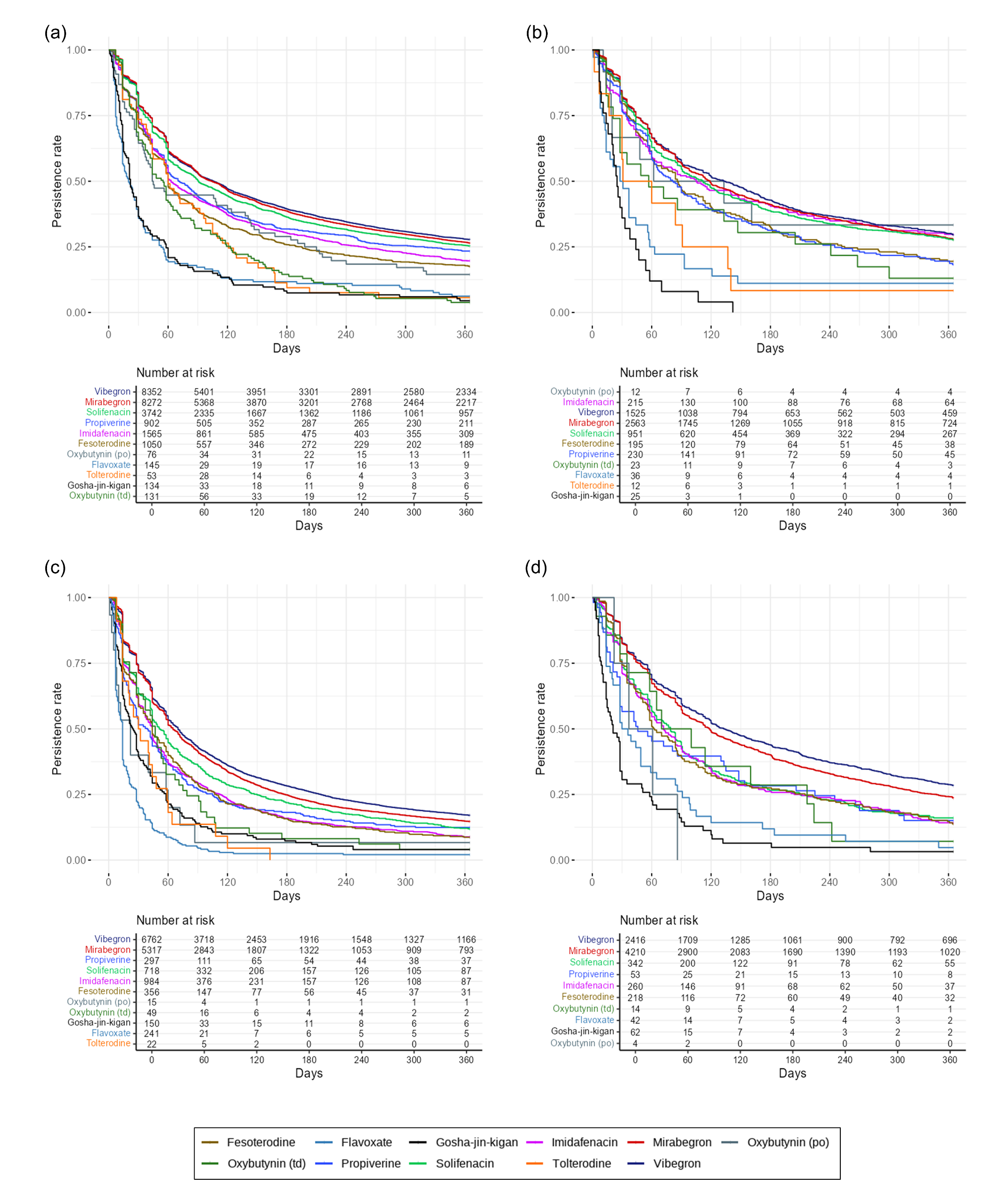

Supplement: Supplementary file 1 — Figure S1: One‐year treatment persistence for individual initial OAB medications. (a) shows 1‐year treatment persistence in internal medicine clinics, (b) in internal medicine hospitals, (c) in urology clinics, and (d) in urology hospitals. Initial treatment was restricted to monotherapy (no concomitant OAB medications on the index date). Abbreviations: OAB, overactive bladder; td, transdermal; po, per os. [file IJU-33-0-s001.docx]
