# Supplementary material for: Prescribing Patterns and Treatment Persistence for Overactive Bladder in Japan Across Medical Specialties and Facility Types: A Nationwide Claims Database Study
Source: Int J Urol. 2026 Apr 21;33:e70468. doi: 10.1111/iju.70468 (PMC13098471; doi:10.1111/iju.70468)
Supplement: Supplementary file 2 — Table S1: Definition of OAB diagnosis and OAB medications. Abbreviations: OAB, Overactive bladder; ATC, Anatomical Therapeutic Chemical Classification System. Table S2: Definition of Comorbidities and tests. Table S3: List of comorbidities defined by ICD‐10 diagnosis codes and ATC codes. Baseline comorbidities by medical specialty and facility type. Values are presented as n (%), with percentages calculated using the total number of patients in each column as the denominator. Comorbidities were defined using a claims‐based algorithm requiring both an ICD‐10 diagnosis code and a corresponding medication record during the 1‐year look‐back period (see Table S2 for code definitions). Dry mouth/xerostomia was not assessed using this combined diagnosis–medication definition because no established definition based on both diagnosis and medication codes has been reported in prior studies; therefore, results are shown as N/A. Table S4: Initial prescription of OAB medications by medical specialty and facility type, stratified by sex. Initial pharmacotherapy for OAB by medical specialty, facility type, and sex. Values are presented as n (%). β3‐adrenoceptor agonists, anticholinergics, and other drugs were defined as described in Table S1. Multiple indicates concomitant prescriptions of ≥ 2 OAB medications on the index date. Abbreviations: OAB, overactive bladder. Table S5: Proportion of initial OAB medications by drug across medical specialties, facility types, and sex. Values are presented as n (%) and represent the distribution of initial OAB pharmacotherapy by drug, stratified by medical specialty, facility type, and sex. Percentages are calculated using the total number of patients in each column as the denominator. Multiple indicates concomitant prescriptions of ≥ 2 OAB medications on the index date. Abbreviations: OAB, overactive bladder; td, transdermal; po, per os. Table S6: Baseline comorbidities by initial OAB medication, stratified by medical specialty and facility [file IJU-33-0-s002.docx]

Supplementary Table S1. Definition of OAB diagnosis and OAB medications. Abbreviations: OAB, Overactive bladder; ATC, Anatomical Therapeutic Chemical Classification System.

| **Variables** | **Codes** | **Descriptions** | |
| --- | --- | --- | --- |
| OAB diagnosis | Diagnosis code: 8844583, 8843031 | Non-neurogenic, OAB | |
| **Variables** | **Codes** | **Drug class** | **Ingredient** |
| OAB medications | ATC-code: G04D4 (Urinary incontinence products) | Anticholinergics | Imidafenacin (OD Tablet 0.1mg, Tablet 0.1mg), Oxybutynin hydrochloride (Transdermal patch 73.5mg, Tablet 1mg/2mg/3mg), Solifenacin succinate (OD Tablet 2.5mg/5mg, Tablet 2.5mg/5mg), Tolterodine tartrate (Capsule 2mg/4mg), Fesoterodine fumarate (Tablet 4mg/8mg), Propiverine hydrochloride (Fine granule 2%, Tablet 10mg/20mg) |
|  |  | β3-adrenoceptor agonists | Vibegron (Tablet 50mg), Mirabegron (Tablet 25mg/50mg) |
|  |  | Other drugs | Flavoxate hydrochloride (Tablet 200mg) |
|  | ATC-code: V03B1 (Kampo medicine) | Other drugs | Gosha-jinki-gan |

Supplementary Table S2. Definition of Comorbidities and tests.

| **Comorbidities** | **ICD-10 codes** | **ATC codes** |
| --- | --- | --- |
| Hypertension [1,2,14] | I10-I15 (Hypertensive diseases) | C02, C03, C07, C08, C09 (Antihypertensives, Diuretics, Beta blocking agents, Calcium channel blockers, Agents acting on the renin-angiotensin system) |
| Diabetes Mellitus [1,2,3,4] | E11-E14 (Diabetes mellitus, excluding Type 1) | A10 (Drugs used in diabetes) |
| Dyslipidemia [5,14] | E78 (Disorders of lipoprotein metabolism and other lipidemias) | C10 (Lipid regulating and anti-atheroma preparations) |
| Dementia [6,7] | F00-F03 (Dementia) G30 (Alzheimer's disease) | N07D (Anti-Alzheimer products) * |
| Glaucoma [8] | H40-H42 (Glaucoma) Q15.0 (Congenital glaucoma) | S01E (Antiglaucoma preparations and miotics) * |
| Benign Prostatic Hyperplasia [9,15] | N40 (Benign prostatic hyperplasia) | G04C (Drugs used in benign prostatic hypertrophy) |
| Dry Mouth / Xerostomia [10] | M35.0 (Sjögren syndrome) R63.1 (Polydipsia) * R68.2 (Dry mouth, unspecified) * | N/A |
| Cerebrovascular Diseases (Stroke) [2,4,11,16,17] | I60-I66 (Cerebrovascular diseases) G45-G46 (Transient cerebral ischemic attacks and related syndromes) | B01 (Antithrombotic agents) |
| Myocardial Infarction [3,4,11,17] | I21-I23 (Acute myocardial infarction) | B01 (Antithrombotic agents) |
| Heart Failure [2,11,12,18] | I50 (Heart failure) I11.0 (Hypertensive heart disease with heart failure) | C01A (Cardiac glycosides and combinations) C03 (Diuretics) |
| Angina Pectoris [4,12,18] | I20 (Angina pectoris) | B01 (Antithrombotic agents) C01E (Nitrites and nitrates) |
| Atrial Fibrillation / Atrial Flutter* [6,11,12,16,19] | I48 (Atrial fibrillation and flutter) I49.0 (Ventricular fibrillation and flutter) | B01 (Antithrombotic agents) |
| Arrhythmia [13,20] | I45 (Other conduction disorders) I47 (Paroxysmal tachycardia) * I49 (Other cardiac arrhythmias) | C01B (Antiarrhythmics, class I and III) |
| **Urological tests** | **Japanese medical procedure codes** | |
| Urinalysis* | 160000310, 160000410, 160001310, 160001410, 160001710, 160003550, 160003750, 160003910, 160004110, 160004310, 160004510, 160004610, 160004810, 160111710, 160111810, 160112010, 160112110, 160112210, 160112310, 160134350,160156710, 160157750, 160169250, 160176350, 160181950, 160189250, 160189950, 160209650, 160210610, 160224650, 160237350, 160005010, 160159550 | |
| Postvoid residual measurements * | 160170310, 160186710, 160072210 (Thoracoabdominal ultrasonography) | |

Reference:

1. Takeuchi M, Shinozaki T, Kawakami K. Universal Health Checkups and Risk of Incident Diabetes and Hypertension. JAMA Netw Open. 2024 Dec 2;7(12):e2451813.

2. Enomoto A, Mano Y, Kawano Y, Nishikawa T, Aoyama T, Sasaki Y, et al. Comparison of the Safety and Effectiveness of Four Direct Oral Anticoagulants in Japanese Patients with Nonvalvular Atrial Fibrillation Using Real-World Data. Biol Pharm Bull. 2021;44(9):1294-1302.

3. Ando T, Hasegawa T, Ishiguro C, Komiyama J, Kuno T, Iwagami M. Difference in outcome event coverage between insurance-based and hospital-based databases: a methodological study of diabetes drug use and cardiovascular events in Japan. Front Pharmacol. 2025 Sep 16;16:1642522.

4. Liu N, Fujino Y, Fujimoto K, Ohtani M, Imamura H, Matsuda S. High-frequency HbA1c testing among older patients with diabetes in Japan: a longitudinal analysis using medical claims data. Diabetol Int. 2022 May 9;13(4):644-656.

5. Sekine A, Nakajima K. Agreement in All-in-One Dataset between Diagnosis and Prescribed Medication for Common Cardiometabolic Diseases in the NDB-K7Ps. Epidemiologia (Basel). 2023 Oct 2;4(4):370-381.

6. Matsunaga M, Tanihara S, He Y, Yatsuya H, Ota A. Sex-specific association of comorbid heart failure on mortality after Alzheimer's disease diagnosis in older adults aged 75 years and above: A health insurance claims data analysis in Japan. J Alzheimers Dis. 2025 Feb;103(3):749-757.

7. Nakaoku Y, Takahashi Y, Tominari S, Nakayama T. Predictors of New Dementia Diagnoses in Elderly Individuals: A Retrospective Cohort Study Based on Prefecture-Wide Claims Data in Japan. Int J Environ Res Public Health. 2021 Jan 13;18(2):629.

8. Fujita A, Hashimoto Y, Matsui H, Yasunaga H, Aihara M. Association between lifestyle habits and glaucoma incidence: a retrospective cohort study. Eye (Lond). 2023 Nov;37(16):3470-3476.

9. Lee YJ, Lee JW, Park J, Seo SI, Chung JI, Yoo TK, Son H. Nationwide incidence and treatment pattern of benign prostatic hyperplasia in Korea. Investig Clin Urol. 2016 Nov;57(6):424-430.

10. Lee CK, Tsai CP, Liao TL, Huang WN, Chen YH, Lin CH, et al. Overactive bladder and bladder pain syndrome/interstitial cystitis in primary Sjögren's syndrome patients: A nationwide population-based study. PLoS One. 2019 Nov 20;14(11):e0225455.

11. Zhang L, Ono Y, Qiao Q, Nagai T. Trends in heart failure prevalence in Japan 2014-2019: a report from healthcare administration databases. ESC Heart Fail. 2023 Jun;10(3):1996-2009.

12. Moribe T, Xu L, Take K, Yonemoto N, Suzuki K. Real-world treatment trends and triple class exposed status in newly diagnosed multiple myeloma patients in Japan: A retrospective claims database study. PLoS One. 2024 Sep 30;19(9):e0310333.

13. Miyamoto K, Murata S, Takegami M, Nakajima K, Kamakura T, Wada M, et al. Real-world comparison of in-hospital complications after catheter ablation for atrial fibrillation between non-antivitamin K anticoagulants and warfarin: A propensity-matched analysis using nation-wide database. Int J Cardiol Heart Vasc. 2023 Jan 11;44:101174.

14. Degli Esposti L, Sangiorgi D, Buda S, Degli Esposti E, Scaglione F. Therapy discontinuation or substitution in patients with cardiovascular disease, switching among different products of the same off-patent active substance: a 'real-world' retrospective cohort study. BMJ Open. 2016 Nov 2;6(11): e012003.

15. Laanani M, Weill A, Jollant F, Zureik M, Dray-Spira R. Suicidal risk associated with finasteride versus dutasteride among men treated for benign prostatic hyperplasia: nationwide cohort study. Sci Rep. 2023 Mar 31;13(1):5308.

16. Huang JA, Lin CH, Wu MJ, Chen YH, Chang KC, Hou CW. Ten-year follow-up investigation of stroke risk in systemic lupus erythematosus. Stroke Vasc Neurol. 2024 Feb 27;9(1):1-7.

17. Umeda T, Hayashi A, Fujimoto G, Piao Y, Matsui N, Tokita S. Medication Adherence/Persistence and Demographics of Japanese Dyslipidemia Patients on Statin-Ezetimibe as a Separate Pill Combination Lipid-Lowering Therapy　- An Observational Pharmacy Claims Database Study. Circ J. 2019 Jul 25;83(8):1689-1697.

18. Vegter S, Nguyen NH, Visser ST, de Jong-van den Berg LT, Postma MJ, Boersma C. Compliance, persistence, and switching patterns for ACE inhibitors and ARBs. Am J Manag Care. 2011 Sep;17(9):609-16.

19. Sabaté M, Vidal X, Ballarin E, Rottenkolber M, Schmiedl S, Grave B, et al. Adherence to Direct Oral Anticoagulants in Patients With Non-Valvular Atrial Fibrillation: A Cross-National Comparison in Six European Countries (2008-2015). Front Pharmacol. 2021 Nov 3;12:682890.

20. Hempenius M, Groenwold RHH, de Boer A, Klungel OH, Gardarsdottir H. Amiodarone use and the risk of acute pancreatitis: Influence of different exposure definitions. Pharmacoepidemiol Drug Saf. 2019 Dec;28(12):1563-1571.

* Selected manually based on clinical judgment.

Supplementary Table S3. List of comorbidities defined by ICD-10 diagnosis codes and ATC codes. Baseline comorbidities by medical specialty and facility type. Values are presented as n (%), with percentages calculated using the total number of patients in each column as the denominator. Comorbidities were defined using a claims-based algorithm requiring both an ICD-10 diagnosis code and a corresponding medication record during the 1-year look-back period (see Supplementary Table S2 for code definitions). Dry mouth/xerostomia was not assessed using this combined diagnosis–medication definition because no established definition based on both diagnosis and medication codes has been reported in prior studies; therefore, results are shown as N/A.

|  | **Overall** | **Internal medicine, Clinic** | **Internal medicine, Hospital** | **Urology, Clinic** | **Urology, Hospital** |
| --- | --- | --- | --- | --- | --- |
|  | **n = 65,173** | **n = 24,751** | **n = 5855** | **n = 15,142** | **n = 7685** |
| **Comorbidity, n (%)** |  |  |  |  |  |
| Hypertension | 42,382 (65.0%) | 17,319 (70.0%) | 4198 (71.7%) | 8236 (54.4%) | 4810 (62.6%) |
| Type 2 diabetes | 11,198 (17.2%) | 4300 (17.4%) | 1444 (24.7%) | 2222 (14.7%) | 1398 (18.2%) |
| Dyslipidemia | 27,462 (42.1%) | 11,234 (45.4%) | 2582 (44.1%) | 5649 (37.3%) | 3032 (39.5%) |
| Dementia | 3917 (6.0%) | 1697 (6.9%) | 473 (8.1%) | 571 (3.8%) | 279 (3.6%) |
| Glaucoma | 6211 (9.5%) | 2408 (9.7%) | 566 (9.7%) | 1379 (9.1%) | 764 (9.9%) |
| Dry mouth/Xerostomia | N/A | N/A | N/A | N/A | N/A |
| Constipation | 24,150 (37.1%) | 8978 (36.3%) | 2763 (47.2%) | 4555 (30.1%) | 2903 (37.8%) |
| Benign prostatic hyperplasia | 15,961 (24.5%) | 3543 (14.3%) | 1379 (23.6%) | 5609 (37.0%) | 3515 (45.7%) |
| Cerebrovascular diseases (Stroke) | 8762 (13.4%) | 3004 (12.1%) | 1013 (17.3%) | 1743 (11.5%) | 1047 (13.6%) |
| Myocardial infarction | 813 (1.2%) | 284 (1.1%) | 87 (1.5%) | 165 (1.1%) | 133 (1.7%) |
| Heart failure | 395 (0.6%) | 154 (0.6%) | 48 (0.8%) | 68 (0.4%) | 43 (0.6%) |
| Angina pectoris | 7866 (12.1%) | 2701 (10.9%) | 935 (16.0%) | 1544 (10.2%) | 1088 (14.2%) |
| Atrial fibrillation/Flutter | 4850 (7.4%) | 1684 (6.8%) | 536 (9.2%) | 1017 (6.7%) | 617 (8.0%) |
| Arrhythmia | 1011 (1.6%) | 343 (1.4%) | 103 (1.8%) | 220 (1.5%) | 129 (1.7%) |

Supplementary Table S4. Initial prescription of OAB medications by medical specialty and facility type, stratified by sex. Initial pharmacotherapy for OAB by medical specialty, facility type, and sex. Values are presented as n (%). β3-adrenoceptor agonists, anticholinergics, and other drugs were defined as described in Supplementary Table S1. Multiple indicates concomitant prescriptions of ≥2 OAB medications on the index date. Abbreviations: OAB, overactive bladder.

|  | **Overall** | | **Internal medicine, Clinic** | | **Internal medicine, Hospital** | | **Urology, Clinic** | | **Urology, Hospital** | | |
| --- | --- | --- | --- | --- | --- | --- | --- | --- | --- | --- | --- |
|  | **Female** | **Male** | **Female** | **Male** | **Female** | **Male** | **Female** | **Male** | **Female** | **Male** |  |
|  | **(n = 36,669)** | **(n = 28,504)** | **(n = 16,258)** | **(n = 8493)** | **(n = 3234)** | **(n = 2621)** | **(n = 7228)** | **(n = 7914)** | **(n = 2337)** | **(n = 5348)** |  |
| β3-adrenoceptor agonists | 24,573 (67.0%) | 22,345 (78.4%) | 10,422 (64.1%) | 6202 (73.0%) | 2092 (64.7%) | 1996 (76.2%) | 5552 (76.8%) | 6527 (82.5%) | 1944 (83.2%) | 4682 (87.5%) |  |
| Anticholinergics | 10,885 (29.7%) | 5503 (19.3%) | 5420 (33.3%) | 2099 (24.7%) | 1062 (32.8%) | 576 (22.0%) | 1274 (17.6%) | 1167 (14.7%) | 311 (13.3%) | 580 (10.8%) |  |
| Other drugs | 663 (1.8%) | 342 (1.2%) | 196 (1.2%) | 83 (1.0%) | 40 (1.2%) | 21 (0.8%) | 249 (3.4%) | 142 (1.8%) | 54 (2.3%) | 50 (0.9%) |  |
| Multiple | 548 (1.5%) | 314 (1.1%) | 220 (1.4%) | 109 (1.3%) | 40 (1.2%) | 28 (1.1%) | 153 (2.1%) | 78 (1.0%) | 28 (1.2%) | 36 (0.7%) |  |

Supplementary Table S5. Proportion of initial OAB medications by drug across medical specialties, facility types, and sex. Values are presented as n (%) and represent the distribution of initial OAB pharmacotherapy by drug, stratified by medical specialty, facility type, and sex. Percentages are calculated using the total number of patients in each column as the denominator. Multiple indicates concomitant prescriptions of ≥2 OAB medications on the index date. Abbreviations: OAB, overactive bladder; td, transdermal; po, per os.

|  | **Overall** | | **Internal medicine, Clinic** | | **Internal medicine, Hospital** | | **Urology, Clinic** | | | **Urology, Hospital** | |
| --- | --- | --- | --- | --- | --- | --- | --- | --- | --- | --- | --- |
|  | **Female** | **Male** | **Female** | **Male** | **Female** | **Male** | | **Female** | **Male** | **Female** | **Male** |
|  | **(n = 36,669)** | **(n = 28,504)** | **(n = 16,258)** | **(n = 8493)** | **(n = 3234)** | **(n = 2621)** | | **(n = 7228)** | **(n = 7914)** | **(n = 2337)** | **(n = 5348)** |
| Mirabegron | 12,928 (35.3%) | 11,781 (41.3%) | 5303 (32.6%) | 2969 (35.0%) | 1379 (42.6%) | 1184 (45.2%) | | 2337 (32.3%) | 2980 (37.7%) | 1241 (53.1%) | 2969 (55.5%) |
| Vibegron | 11,645 (31.8%) | 10,564 (37.1%) | 5119 (31.5%) | 3233 (38.1%) | 713 (22.0%) | 812 (31.0%) | | 3215 (44.5%) | 3547 (44.8%) | 703 (30.1%) | 1713 (32.0%) |
| Solifenacin | 5329 (14.5%) | 2383 (8.4%) | 2746 (16.9%) | 996 (11.7%) | 622 (19.2%) | 329 (12.6%) | | 381 (5.3%) | 337 (4.3%) | 123 (5.3%) | 219 (4.1%) |
| Imidafenacin | 2248 (6.1%) | 1485 (5.2%) | 1095 (6.7%) | 470 (5.5%) | 127 (3.9%) | 88 (3.4%) | | 469 (6.5%) | 515 (6.5%) | 79 (3.4%) | 181 (3.4%) |
| Fesoterodine | 1499 (4.1%) | 779 (2.7%) | 756 (4.7%) | 294 (3.5%) | 124 (3.8%) | 71 (2.7%) | | 203 (2.8%) | 153 (1.9%) | 82 (3.5%) | 136 (2.5%) |
| Propiverine | 1392 (3.8%) | 673 (2.4%) | 630 (3.9%) | 272 (3.2%) | 161 (5.0%) | 69 (2.6%) | | 181 (2.5%) | 116 (1.5%) | 22 (0.9%) | 31 (0.6%) |
| Flavoxate | 389 (1.1%) | 158 (0.6%) | 108 (0.7%) | 37 (0.4%) | 30 (0.9%) | 6 (0.2%) | | 164 (2.3%) | 77 (1.0%) | 23 (1.0%) | 19 (0.4%) |
| Gosha-jinki-gan | 274 (0.7%) | 184 (0.6%) | 88 (0.5%) | 46 (0.5%) | 10 (0.3%) | 15 (0.6%) | | 85 (1.2%) | 65 (0.8%) | 31 (1.3%) | 31 (0.6%) |
| Oxybutynin (td) | 226 (0.6%) | 91 (0.3%) | 101 (0.6%) | 30 (0.4%) | 16 (0.5%) | 7 (0.3%) | | 24 (0.3%) | 25 (0.3%) | 3 (0.1%) | 11 (0.2%) |
| Oxybutynin (po) | 111 (0.3%) | 42 (0.1%) | 60 (0.4%) | 16 (0.2%) | 7 (0.2%) | 5 (0.2%) | | 8 (0.1%) | 7 (0.1%) | 2 (0.1%) | 2 (<0.1%) |
| Tolterodine | 80 (0.2%) | 50 (0.2%) | 32 (0.2%) | 21 (0.2%) | 5 (0.2%) | 7 (0.3%) | | 8 (0.1%) | 14 (0.2%) | 0 (0.0%) | 0 (0.0%) |
| Multiple | 548 (1.5%) | 314 (1.1%) | 220 (1.4%) | 109 (1.3%) | 40 (1.2%) | 28 (1.1%) | | 153 (2.1%) | 78 (1.0%) | 28 (1.2%) | 36 (0.7%) |

Supplementary Table S6. Baseline comorbidities by initial OAB medication, stratified by medical specialty and facility type. Baseline comorbidities stratified by initial OAB medication and care setting. Initial treatment was restricted to monotherapy (no concomitant OAB medications on the index date). Values are presented as n (%), with percentages calculated using the total number of patients who initiated each medication (shown in the column headers) as the denominator. Results are shown for the overall cohort and separately for four specialty–facility groups (Internal medicine, Clinic; Internal medicine, Hospital; Urology, Clinic; and Urology, Hospital). Comorbidities were identified during the 1-year look-back period using ICD-10 diagnosis codes in claims data (see Supplementary Table S2 for code definitions). Cells are shown as N/A when no patients initiated the medication in that subgroup. Abbreviations: OAB, overactive bladder.

|  | **Overall** | | | | | | | | | | |
| --- | --- | --- | --- | --- | --- | --- | --- | --- | --- | --- | --- |
|  | **Mirabegron** | **Vibegron** | **Fesoterodine** | **Imidafenacin** | **Oxybutynin patch** | **Oxybutynin oral** | **Propiverine** | **Solifenacin** | **Tolterodine** | **Flavoxate** | **Gosha-jinki-gan** |
|  | **(n = 24,709)** | **(n = 22,209)** | **(n = 2278)** | **(n = 3733)** | **(n = 317)** | **(n = 153)** | **(n = 2065)** | **(n = 7712)** | **(n = 130)** | **(n = 547)** | **(n = 458)** |
| **Comorbidity, n (%)** |  |  |  |  |  |  |  |  |  |  |  |
| Hypertension | 17,589 (71.2%) | 15,297 (68.9%) | 1557 (68.3%) | 2545 (68.2%) | 243 (76.7%) | 109 (71.2%) | 1486 (72.0%) | 5615 (72.8%) | 87 (66.9%) | 373 (68.2%) | 304 (66.4%) |
| Type 2 diabetes | 9620 (38.9%) | 8409 (37.9%) | 796 (34.9%) | 1325 (35.5%) | 125 (39.4%) | 52 (34.0%) | 710 (34.4%) | 2772 (35.9%) | 49 (37.7%) | 187 (34.2%) | 175 (38.2%) |
| Dyslipidemia | 13,915 (56.3%) | 12,572 (56.6%) | 1262 (55.4%) | 2080 (55.7%) | 177 (55.8%) | 82 (53.6%) | 1155 (55.9%) | 4433 (57.5%) | 74 (56.9%) | 303 (55.4%) | 256 (55.9%) |
| Dementia | 2602 (10.5%) | 1869 (8.4%) | 222 (9.7%) | 368 (9.9%) | 50 (15.8%) | 22 (14.4%) | 306 (14.8%) | 939 (12.2%) | 14 (10.8%) | 52 (9.5%) | 36 (7.9%) |
| Glaucoma | 3616 (14.6%) | 3051 (13.7%) | 258 (11.3%) | 458 (12.3%) | 43 (13.6%) | 22 (14.4%) | 221 (10.7%) | 979 (12.7%) | 12 (9.2%) | 69 (12.6%) | 76 (16.6%) |
| Dry mouth/Xerostomia | 360 (1.5%) | 317 (1.4%) | 38 (1.7%) | 43 (1.2%) | 5 (1.6%) | 4 (2.6%) | 36 (1.7%) | 103 (1.3%) | 3 (2.3%) | 6 (1.1%) | 11 (2.4%) |
| Constipation | 11,664 (47.2%) | 9829 (44.3%) | 1028 (45.1%) | 1581 (42.4%) | 142 (44.8%) | 79 (51.6%) | 1041 (50.4%) | 3741 (48.5%) | 51 (39.2%) | 263 (48.1%) | 229 (50.0%) |
| Benign prostatic hyperplasia | 8453 (34.2%) | 7386 (33.3%) | 426 (18.7%) | 949 (25.4%) | 59 (18.6%) | 19 (12.4%) | 341 (16.5%) | 1197 (15.5%) | 23 (17.7%) | 105 (19.2%) | 138 (30.1%) |
| Cerebrovascular diseases (Stroke) | 5032 (20.4%) | 4449 (20.0%) | 426 (18.7%) | 734 (19.7%) | 68 (21.5%) | 31 (20.3%) | 407 (19.7%) | 1480 (19.2%) | 29 (22.3%) | 96 (17.6%) | 93 (20.3%) |
| Myocardial infarction | 351 (1.4%) | 314 (1.4%) | 31 (1.4%) | 58 (1.6%) | 2 (0.6%) | 2 (1.3%) | 23 (1.1%) | 105 (1.4%) | 4 (3.1%) | 9 (1.6%) | 3 (0.7%) |
| Heart failure | 6316 (25.6%) | 5503 (24.8%) | 583 (25.6%) | 895 (24.0%) | 93 (29.3%) | 35 (22.9%) | 559 (27.1%) | 2005 (26.0%) | 27 (20.8%) | 143 (26.1%) | 121 (26.4%) |
| Angina pectoris | 4607 (18.6%) | 4041 (18.2%) | 397 (17.4%) | 642 (17.2%) | 63 (19.9%) | 24 (15.7%) | 366 (17.7%) | 1401 (18.2%) | 17 (13.1%) | 102 (18.6%) | 96 (21.0%) |
| Atrial fibrillation/Flutter | 2055 (8.3%) | 1971 (8.9%) | 207 (9.1%) | 339 (9.1%) | 33 (10.4%) | 9 (5.9%) | 175 (8.5%) | 654 (8.5%) | 10 (7.7%) | 37 (6.8%) | 47 (10.3%) |
| Arrhythmia | 3252 (13.2%) | 3034 (13.7%) | 289 (12.7%) | 549 (14.7%) | 47 (14.8%) | 23 (15.0%) | 256 (12.4%) | 999 (13.0%) | 15 (11.5%) | 76 (13.9%) | 71 (15.5%) |

|  | **Internal medicine, Clinic** | | | | | | | | | | |
| --- | --- | --- | --- | --- | --- | --- | --- | --- | --- | --- | --- |
|  | **Mirabegron** | **Vibegron** | **Fesoterodine** | **Imidafenacin** | **Oxybutynin patch** | **Oxybutynin oral** | **Propiverine** | **Solifenacin** | **Tolterodine** | **Flavoxate** | **Gosha-jinki-gan** |
|  | **(n = 8272)** | **(n = 8352)** | **(n = 1050)** | **(n = 1565)** | **(n = 131)** | **(n = 76)** | **(n = 902)** | **(n = 3742)** | **(n = 53)** | **(n = 145)** | **(n = 134)** |
| **Comorbidity, n (%)** |  |  |  |  |  |  |  |  |  |  |  |
| Hypertension | 6304 (76.2%) | 6234 (74.6%) | 786 (74.9%) | 1139 (72.8%) | 112 (85.5%) | 54 (71.1%) | 674 (74.7%) | 2889 (77.2%) | 39 (73.6%) | 113 (77.9%) | 85 (63.4%) |
| Type 2 diabetes | 3278 (39.6%) | 3259 (39.0%) | 392 (37.3%) | 568 (36.3%) | 52 (39.7%) | 31 (40.8%) | 323 (35.8%) | 1378 (36.8%) | 23 (43.4%) | 51 (35.2%) | 49 (36.6%) |
| Dyslipidemia | 4911 (59.4%) | 5095 (61.0%) | 643 (61.2%) | 932 (59.6%) | 75 (57.3%) | 41 (53.9%) | 521 (57.8%) | 2303 (61.5%) | 33 (62.3%) | 80 (55.2%) | 76 (56.7%) |
| Dementia | 988 (11.9%) | 826 (9.9%) | 109 (10.4%) | 147 (9.4%) | 22 (16.8%) | 13 (17.1%) | 124 (13.7%) | 466 (12.5%) | 9 (17.0%) | 21 (14.5%) | 13 (9.7%) |
| Glaucoma | 1232 (14.9%) | 1117 (13.4%) | 132 (12.6%) | 186 (11.9%) | 20 (15.3%) | 11 (14.5%) | 100 (11.1%) | 499 (13.3%) | 7 (13.2%) | 19 (13.1%) | 27 (20.1%) |
| Dry mouth/Xerostomia | 128 (1.5%) | 114 (1.4%) | 13 (1.2%) | 15 (1.0%) | 4 (3.1%) | 3 (3.9%) | 14 (1.6%) | 49 (1.3%) | 0 (0.0%) | 1 (0.7%) | 1 (0.7%) |
| Constipation | 3829 (46.3%) | 3707 (44.4%) | 473 (45.0%) | 643 (41.1%) | 57 (43.5%) | 39 (51.3%) | 438 (48.6%) | 1768 (47.2%) | 19 (35.8%) | 82 (56.6%) | 63 (47.0%) |
| Benign prostatic hyperplasia | 1573 (19.0%) | 1722 (20.6%) | 118 (11.2%) | 230 (14.7%) | 19 (14.5%) | 6 (7.9%) | 124 (13.7%) | 434 (11.6%) | 11 (20.8%) | 18 (12.4%) | 31 (23.1%) |
| Cerebrovascular diseases (Stroke) | 1525 (18.4%) | 1672 (20.0%) | 185 (17.6%) | 278 (17.8%) | 32 (24.4%) | 17 (22.4%) | 158 (17.5%) | 689 (18.4%) | 11 (20.8%) | 27 (18.6%) | 25 (18.7%) |
| Myocardial infarction | 96 (1.2%) | 115 (1.4%) | 16 (1.5%) | 22 (1.4%) | 2 (1.5%) | 0 (0.0%) | 10 (1.1%) | 52 (1.4%) | 0 (0.0%) | 1 (0.7%) | 0 (0.0%) |
| Heart failure | 2237 (27.0%) | 2174 (26.0%) | 257 (24.5%) | 376 (24.0%) | 46 (35.1%) | 24 (31.6%) | 236 (26.2%) | 993 (26.5%) | 11 (20.8%) | 40 (27.6%) | 35 (26.1%) |
| Angina pectoris | 1471 (17.8%) | 1520 (18.2%) | 195 (18.6%) | 258 (16.5%) | 27 (20.6%) | 11 (14.5%) | 151 (16.7%) | 654 (17.5%) | 10 (18.9%) | 32 (22.1%) | 23 (17.2%) |
| Atrial fibrillation/Flutter | 646 (7.8%) | 680 (8.1%) | 83 (7.9%) | 118 (7.5%) | 9 (6.9%) | 4 (5.3%) | 66 (7.3%) | 299 (8.0%) | 4 (7.5%) | 8 (5.5%) | 9 (6.7%) |
| Arrhythmia | 1109 (13.4%) | 1178 (14.1%) | 144 (13.7%) | 224 (14.3%) | 24 (18.3%) | 12 (15.8%) | 116 (12.9%) | 480 (12.8%) | 7 (13.2%) | 25 (17.2%) | 20 (14.9%) |

|  | **Internal medicine, Hospital** | | | | | | | | | | |
| --- | --- | --- | --- | --- | --- | --- | --- | --- | --- | --- | --- |
|  | **Mirabegron** | **Vibegron** | **Fesoterodine** | **Imidafenacin** | **Oxybutynin patch** | **Oxybutynin oral** | **Propiverine** | **Solifenacin** | **Tolterodine** | **Flavoxate** | **Gosha-jinki-gan** |
|  | **(n = 2563)** | **(n = 1525)** | **(n = 195)** | **(n = 215)** | **(n = 23)** | **(n = 12)** | **(n = 230)** | **(n = 951)** | **(n = 12)** | **(n = 36)** | **(n = 25)** |
| **Comorbidity, n (%)** |  |  |  |  |  |  |  |  |  |  |  |
| Hypertension | 2008 (78.3%) | 1152 (75.5%) | 141 (72.3%) | 171 (79.5%) | 17 (73.9%) | 10 (83.3%) | 191 (83.0%) | 717 (75.4%) | 10 (83.3%) | 30 (83.3%) | 20 (80.0%) |
| Type 2 diabetes | 1198 (46.7%) | 711 (46.6%) | 79 (40.5%) | 88 (40.9%) | 8 (34.8%) | 4 (33.3%) | 89 (38.7%) | 398 (41.9%) | 4 (33.3%) | 9 (25.0%) | 12 (48.0%) |
| Dyslipidemia | 1515 (59.1%) | 892 (58.5%) | 115 (59.0%) | 120 (55.8%) | 11 (47.8%) | 5 (41.7%) | 127 (55.2%) | 542 (57.0%) | 5 (41.7%) | 19 (52.8%) | 16 (64.0%) |
| Dementia | 384 (15.0%) | 148 (9.7%) | 23 (11.8%) | 40 (18.6%) | 4 (17.4%) | 3 (25.0%) | 58 (25.2%) | 177 (18.6%) | 3 (25.0%) | 6 (16.7%) | 3 (12.0%) |
| Glaucoma | 381 (14.9%) | 222 (14.6%) | 20 (10.3%) | 26 (12.1%) | 3 (13.0%) | 0 (0.0%) | 22 (9.6%) | 117 (12.3%) | 2 (16.7%) | 8 (22.2%) | 7 (28.0%) |
| Dry mouth/Xerostomia | 41 (1.6%) | 31 (2.0%) | 4 (2.1%) | 1 (0.5%) | 0 (0.0%) | 0 (0.0%) | 2 (0.9%) | 11 (1.2%) | 0 (0.0%) | 0 (0.0%) | 0 (0.0%) |
| Constipation | 1459 (56.9%) | 822 (53.9%) | 110 (56.4%) | 127 (59.1%) | 13 (56.5%) | 8 (66.7%) | 150 (65.2%) | 588 (61.8%) | 7 (58.3%) | 21 (58.3%) | 17 (68.0%) |
| Benign prostatic hyperplasia | 825 (32.2%) | 557 (36.5%) | 42 (21.5%) | 52 (24.2%) | 4 (17.4%) | 3 (25.0%) | 32 (13.9%) | 149 (15.7%) | 3 (25.0%) | 4 (11.1%) | 9 (36.0%) |
| Cerebrovascular diseases (Stroke) | 616 (24.0%) | 349 (22.9%) | 48 (24.6%) | 64 (29.8%) | 1 (4.3%) | 2 (16.7%) | 52 (22.6%) | 230 (24.2%) | 2 (16.7%) | 10 (27.8%) | 3 (12.0%) |
| Myocardial infarction | 39 (1.5%) | 19 (1.2%) | 4 (2.1%) | 6 (2.8%) | 0 (0.0%) | 1 (8.3%) | 5 (2.2%) | 14 (1.5%) | 0 (0.0%) | 0 (0.0%) | 1 (4.0%) |
| Heart failure | 861 (33.6%) | 499 (32.7%) | 80 (41.0%) | 74 (34.4%) | 6 (26.1%) | 3 (25.0%) | 84 (36.5%) | 305 (32.1%) | 5 (41.7%) | 10 (27.8%) | 13 (52.0%) |
| Angina pectoris | 592 (23.1%) | 361 (23.7%) | 42 (21.5%) | 45 (20.9%) | 5 (21.7%) | 2 (16.7%) | 58 (25.2%) | 202 (21.2%) | 1 (8.3%) | 8 (22.2%) | 10 (40.0%) |
| Atrial fibrillation/Flutter | 238 (9.3%) | 167 (11.0%) | 25 (12.8%) | 24 (11.2%) | 2 (8.7%) | 0 (0.0%) | 26 (11.3%) | 107 (11.3%) | 1 (8.3%) | 4 (11.1%) | 4 (16.0%) |
| Arrhythmia | 318 (12.4%) | 211 (13.8%) | 23 (11.8%) | 26 (12.1%) | 3 (13.0%) | 2 (16.7%) | 27 (11.7%) | 137 (14.4%) | 2 (16.7%) | 10 (27.8%) | 10 (40.0%) |

|  | **Urology, Clinic** | | | | | | | | | | |
| --- | --- | --- | --- | --- | --- | --- | --- | --- | --- | --- | --- |
|  | **Mirabegron** | **Vibegron** | **Fesoterodine** | **Imidafenacin** | **Oxybutynin patch** | **Oxybutynin oral** | **Propiverine** | **Solifenacin** | **Tolterodine** | **Flavoxate** | **Gosha-jinki-gan** |
|  | **(n = 5317)** | **(n = 6762)** | **(n = 356)** | **(n = 984)** | **(n = 49)** | **(n = 15)** | **(n = 297)** | **(n = 718)** | **(n = 22)** | **(n = 241)** | **(n = 150)** |
| **Comorbidity, n (%)** |  |  |  |  |  |  |  |  |  |  |  |
| Hypertension | 3298 (62.0%) | 4003 (59.2%) | 186 (52.2%) | 572 (58.1%) | 33 (67.3%) | 9 (60.0%) | 173 (58.2%) | 376 (52.4%) | 15 (68.2%) | 150 (62.2%) | 92 (61.3%) |
| Type 2 diabetes | 1806 (34.0%) | 2343 (34.6%) | 104 (29.2%) | 330 (33.5%) | 17 (34.7%) | 5 (33.3%) | 90 (30.3%) | 211 (29.4%) | 7 (31.8%) | 71 (29.5%) | 41 (27.3%) |
| Dyslipidemia | 2803 (52.7%) | 3435 (50.8%) | 157 (44.1%) | 495 (50.3%) | 25 (51.0%) | 8 (53.3%) | 147 (49.5%) | 338 (47.1%) | 13 (59.1%) | 133 (55.2%) | 66 (44.0%) |
| Dementia | 339 (6.4%) | 415 (6.1%) | 24 (6.7%) | 76 (7.7%) | 8 (16.3%) | 2 (13.3%) | 21 (7.1%) | 33 (4.6%) | 1 (4.5%) | 13 (5.4%) | 9 (6.0%) |
| Glaucoma | 795 (15.0%) | 909 (13.4%) | 24 (6.7%) | 116 (11.8%) | 6 (12.2%) | 3 (20.0%) | 35 (11.8%) | 64 (8.9%) | 1 (4.5%) | 25 (10.4%) | 17 (11.3%) |
| Dry mouth/Xerostomia | 73 (1.4%) | 88 (1.3%) | 4 (1.1%) | 11 (1.1%) | 0 (0.0%) | 0 (0.0%) | 6 (2.0%) | 6 (0.8%) | 0 (0.0%) | 3 (1.2%) | 2 (1.3%) |
| Constipation | 2116 (39.8%) | 2616 (38.7%) | 132 (37.1%) | 362 (36.8%) | 29 (59.2%) | 8 (53.3%) | 103 (34.7%) | 235 (32.7%) | 3 (13.6%) | 93 (38.6%) | 61 (40.7%) |
| Benign prostatic hyperplasia | 2634 (49.5%) | 2974 (44.0%) | 98 (27.5%) | 403 (41.0%) | 19 (38.8%) | 5 (33.3%) | 83 (27.9%) | 259 (36.1%) | 9 (40.9%) | 60 (24.9%) | 53 (35.3%) |
| Cerebrovascular diseases (Stroke) | 1000 (18.8%) | 1212 (17.9%) | 52 (14.6%) | 168 (17.1%) | 13 (26.5%) | 0 (0.0%) | 51 (17.2%) | 93 (13.0%) | 4 (18.2%) | 37 (15.4%) | 24 (16.0%) |
| Myocardial infarction | 58 (1.1%) | 98 (1.4%) | 2 (0.6%) | 10 (1.0%) | 0 (0.0%) | 0 (0.0%) | 3 (1.0%) | 8 (1.1%) | 3 (13.6%) | 5 (2.1%) | 1 (0.7%) |
| Heart failure | 997 (18.8%) | 1401 (20.7%) | 60 (16.9%) | 201 (20.4%) | 17 (34.7%) | 1 (6.7%) | 63 (21.2%) | 137 (19.1%) | 6 (27.3%) | 62 (25.7%) | 25 (16.7%) |
| Angina pectoris | 823 (15.5%) | 1061 (15.7%) | 46 (12.9%) | 167 (17.0%) | 11 (22.4%) | 3 (20.0%) | 44 (14.8%) | 104 (14.5%) | 3 (13.6%) | 37 (15.4%) | 22 (14.7%) |
| Atrial fibrillation/Flutter | 393 (7.4%) | 560 (8.3%) | 25 (7.0%) | 99 (10.1%) | 8 (16.3%) | 2 (13.3%) | 26 (8.8%) | 50 (7.0%) | 2 (9.1%) | 14 (5.8%) | 14 (9.3%) |
| Arrhythmia | 658 (12.4%) | 833 (12.3%) | 38 (10.7%) | 144 (14.6%) | 7 (14.3%) | 1 (6.7%) | 44 (14.8%) | 92 (12.8%) | 3 (13.6%) | 34 (14.1%) | 21 (14.0%) |

|  | **Urology, Hospital** | | | | | | | | | | |
| --- | --- | --- | --- | --- | --- | --- | --- | --- | --- | --- | --- |
|  | **Mirabegron** | **Vibegron** | **Fesoterodine** | **Imidafenacin** | **Oxybutynin patch** | **Oxybutynin oral** | **Propiverine** | **Solifenacin** | **Tolterodine** | **Flavoxate** | **Gosha-jinki-gan** |
|  | **(n = 4210)** | **(n = 2416)** | **(n = 218)** | **(n = 260)** | **(n = 14)** | **(n = 4)** | **(n = 53)** | **(n = 342)** | **(n = 0)** | **(n = 42)** | **(n = 62)** |
| **Comorbidity, n (%)** |  |  |  |  |  |  |  |  |  |  |  |
| Hypertension | 2833 (67.3%) | 1666 (69.0%) | 131 (60.1%) | 177 (68.1%) | 7 (50.0%) | 3 (75.0%) | 28 (52.8%) | 230 (67.3%) | N/A | 24 (57.1%) | 45 (72.6%) |
| Type 2 diabetes | 1715 (40.7%) | 942 (39.0%) | 64 (29.4%) | 101 (38.8%) | 8 (57.1%) | 2 (50.0%) | 14 (26.4%) | 136 (39.8%) | N/A | 15 (35.7%) | 30 (48.4%) |
| Dyslipidemia | 2207 (52.4%) | 1313 (54.3%) | 97 (44.5%) | 151 (58.1%) | 7 (50.0%) | 2 (50.0%) | 23 (43.4%) | 180 (52.6%) | N/A | 25 (59.5%) | 41 (66.1%) |
| Dementia | 289 (6.9%) | 146 (6.0%) | 10 (4.6%) | 10 (3.8%) | 0 (0.0%) | 0 (0.0%) | 3 (5.7%) | 17 (5.0%) | N/A | 3 (7.1%) | 3 (4.8%) |
| Glaucoma | 589 (14.0%) | 367 (15.2%) | 29 (13.3%) | 39 (15.0%) | 1 (7.1%) | 1 (25.0%) | 5 (9.4%) | 41 (12.0%) | N/A | 6 (14.3%) | 10 (16.1%) |
| Dry mouth/Xerostomia | 42 (1.0%) | 35 (1.4%) | 5 (2.3%) | 8 (3.1%) | 0 (0.0%) | 0 (0.0%) | 0 (0.0%) | 3 (0.9%) | N/A | 0 (0.0%) | 2 (3.2%) |
| Constipation | 1950 (46.3%) | 1157 (47.9%) | 91 (41.7%) | 125 (48.1%) | 7 (50.0%) | 2 (50.0%) | 26 (49.1%) | 150 (43.9%) | N/A | 18 (42.9%) | 37 (59.7%) |
| Benign prostatic hyperplasia | 2401 (57.0%) | 1359 (56.3%) | 103 (47.2%) | 144 (55.4%) | 7 (50.0%) | 1 (25.0%) | 21 (39.6%) | 154 (45.0%) | N/A | 13 (31.0%) | 28 (45.2%) |
| Cerebrovascular diseases (Stroke) | 803 (19.1%) | 491 (20.3%) | 43 (19.7%) | 50 (19.2%) | 3 (21.4%) | 1 (25.0%) | 4 (7.5%) | 58 (17.0%) | N/A | 6 (14.3%) | 16 (25.8%) |
| Myocardial infarction | 87 (2.1%) | 44 (1.8%) | 2 (0.9%) | 4 (1.5%) | 0 (0.0%) | 0 (0.0%) | 0 (0.0%) | 7 (2.0%) | N/A | 1 (2.4%) | 0 (0.0%) |
| Heart failure | 1014 (24.1%) | 564 (23.3%) | 61 (28.0%) | 72 (27.7%) | 5 (35.7%) | 2 (50.0%) | 10 (18.9%) | 93 (27.2%) | N/A | 10 (23.8%) | 23 (37.1%) |
| Angina pectoris | 849 (20.2%) | 472 (19.5%) | 40 (18.3%) | 68 (26.2%) | 6 (42.9%) | 1 (25.0%) | 4 (7.5%) | 71 (20.8%) | N/A | 8 (19.0%) | 20 (32.3%) |
| Atrial fibrillation/Flutter | 346 (8.2%) | 230 (9.5%) | 37 (17.0%) | 29 (11.2%) | 2 (14.3%) | 1 (25.0%) | 2 (3.8%) | 34 (9.9%) | N/A | 4 (9.5%) | 9 (14.5%) |
| Arrhythmia | 545 (12.9%) | 349 (14.4%) | 25 (11.5%) | 48 (18.5%) | 0 (0.0%) | 1 (25.0%) | 9 (17.0%) | 50 (14.6%) | N/A | 3 (7.1%) | 7 (11.3%) |

Supplementary Table S7. Concomitant BPH medications among patients with BPH, stratified by medical specialty and facility type.

|  | **Overall** | **Internal medicine, Clinic** | **Internal medicine, Hospital** | **Urology, Clinic** | **Urology, Hospital** | |
| --- | --- | --- | --- | --- | --- | --- |
|  | **(n = 19,298)** | **(n = 4349)** | **(n = 1694)** | **(n = 6660)** | **(n = 4256)** | |
| α1-Adrenoceptor antagonists | 14,807 (76.7%) | 3,344 (76.9%) | 1,305 (77.0%) | 5,071 (76.1%) | 3,291 (77.3%) |  |
| 5α-Reductase inhibitors | 2,007 (10.4%) | 396 (9.1%) | 139 (8.2%) | 749 (11.2%) | 487 (11.4%) |  |
| PDE5 inhibitors | 1,590 (8.2%) | 224 (5.2%) | 108 (6.4%) | 761 (11.4%) | 357 (8.4%) |  |
| Antiandrogens | 120 (0.6%) | 35 (0.8%) | 7 (0.4%) | 39 (0.6%) | 25 (0.6%) |  |
| Chimaphila umbellata ext., Populus tremula ext., Pulsatilla pratensis mill ext., Equisetum arvense ext., Wheat Germ Oil | 758 (3.9%) | 153 (3.5%) | 61 (3.6%) | 282 (4.2%) | 180 (4.2%) |  |
| Cernitin pollen extract | 903 (4.7%) | 140 (3.2%) | 51 (3.0%) | 484 (7.3%) | 156 (3.7%) |  |

Supplementary Table S8. Persistence rates of initial OAB medications at Days 90, 180, and 365, by medical specialty and facility type. Persistence was estimated using the Kaplan–Meier method. Values are treatment persistence estimates (%). n indicates the number of patients at baseline; n in parentheses indicates the number at risk at each time point. N/A, not estimable because no patients were at risk at the time point. Abbreviations: OAB, overactive bladder; td, transdermal; po, per os.

| **Internal medicine, Clinic** | **Vibegron  (n = 8352)** | **Mirabegron  (n = 8272)** | **Solifenacin  (n = 3742)** | **Propiverine  (n = 902)** | **Imidafenacin  (n = 1565)** | **Fesoterodine  (n = 1050)** | **Oxybutynin (po) (n = 76)** | **Flavoxate  (n = 145)** | **Tolterodine  (n = 53)** | **Gosha-jin-kigan  (n = 134)** | **Oxybutynin (td)  (n = 131)** |
| --- | --- | --- | --- | --- | --- | --- | --- | --- | --- | --- | --- |
| Day 90 | 52.8%  (n = 4452) | 53.0%  (n = 4429) | 50.1%  (n = 1896) | 44.1%  (n = 402) | 43.1%  (n = 680) | 38.4%  (n = 409) | 44.7%  (n = 34) | 17.2%  (n = 25) | 39.6%  (n = 21) | 15.7%  (n = 21) | 32.1%  (n = 42) |
| Day 180 | 39.4%  (n = 3301) | 38.6%  (n = 3201) | 36.3%  (n = 1362) | 31.8%  (n = 287) | 30.2%  (n = 475) | 25.8%  (n = 272) | 28.9%  (n = 22) | 11.7%  (n = 17) | 9.4%  (n = 6) | 7.5%  (n = 11) | 14.5%  (n = 19) |
| Day 365 | 27.8%  (n = 2321) | 26.4%  (n = 2185) | 25.3%  (n = 948) | 23.4%  (n = 211) | 19.7%  (n = 308) | 17.5%  (n = 184) | 14.5%  (n = 11) | 6.2%  (n = 9) | 5.7%  (n = 3) | 4.5%  (n = 6) | 3.8%  (n = 5) |

| **Internal medicine, Hospital** | **Oxybutynin (po)  (n = 12)** | **Imidafenacin  (n = 215)** | **Vibegron  (n = 1525)** | **Mirabegron  (n = 2563)** | **Solifenacin  (n = 951)** | **Fesoterodine  (n = 195)** | **Propiverine  (n = 230)** | **Oxybutynin (td)  (n = 23)** | **Flavoxate  (n = 36)** | **Tolterodine  (n = 12)** | **Gosha-jin-kigan  (n = 25)** |
| --- | --- | --- | --- | --- | --- | --- | --- | --- | --- | --- | --- |
| Day 90 | 50.0%  (n = 6) | 52.1%  (n = 112) | 57.0%  (n = 887) | 56.6%  (n = 1465) | 54.6%  (n = 530) | 46.7%  (n = 93) | 45.7%  (n = 110) | 39.1%  (n = 9) | 22.2%  (n = 8) | 33.3%  (n = 4) | 8.0%  (n = 2) |
| Day 180 | 33.3%  (n = 4) | 40.5%  (n = 88) | 42.8%  (n = 653) | 41.1%  (n = 1055) | 38.6%  (n = 369) | 32.3%  (n = 64) | 31.3%  (n = 72) | 30.4%  (n = 7) | 11.1%  (n = 4) | 8.3%  (n = 1) | N/A |
| Day 365 | 33.3%  (n = 4) | 29.8%  (n = 64) | 29.4%  (n = 451) | 27.9%  (n = 715) | 27.5%  (n = 263) | 19.5%  (n = 38) | 18.3%  (n = 42) | 13.0%  (n = 3) | 11.1%  (n = 4) | 8.3%  (n = 1) | N/A |

| **Urology, Clinic** | **Vibegron (n = 6762)** | **Mirabegron (n = 5317)** | **Propiverine (n = 297)** | **Solifenacin (n = 718)** | **Imidafenacin  (n = 984)** | **Fesoterodine (n = 356)** | **Oxybutynin (po) (n = 15)** | **Oxybutynin (td) (n = 49)** | **Gosha-jin-kigan (n = 150)** | **Flavoxate (n = 241)** | **Tolterodine (n = 22)** |
| --- | --- | --- | --- | --- | --- | --- | --- | --- | --- | --- | --- |
| Day 90 | 42.9% (n = 2934) | 41.2%  (n = 2219) | 26.9% (n = 81) | 35.9% (n = 262) | 29.3% (n = 291) | 28.7% (n = 105) | 6.7% (n = 1) | 24.5% (n = 12) | 14.0% (n = 21) | 4.6% (n = 12) | 13.6% (n = 3) |
| Day 180 | 28.2% (n = 1916) | 24.8% (n = 1322) | 18.2% (n = 54) | 21.9% (n = 157) | 16.0% (n = 157) | 15.7% (n = 56) | 6.7% (n = 1) | 8.2% (n = 4) | 7.3% (n = 11) | 2.5% (n = 6) | N/A |
| Day 365 | 17.0% (n = 1152) | 14.7% (n = 781) | 12.5% (n = 37) | 11.8% (n = 85) | 8.8% (n = 87) | 8.7% (n = 31) | 6.7% (n = 1) | 4.1% (n = 2) | 4.0% (n = 6) | 2.1% (n = 5) | N/A |

| **Urology, Hospital** | **Vibegron  (n = 2416)** | **Mirabegron  (n = 4210)** | **Solifenacin (n = 342)** | **Propiverine  (n = 53)** | **Imidafenacin  (n = 260)** | **Fesoterodine  (n = 218)** | **Oxybutynin (td) (n = 14)** | **Flavoxate  (n = 42)** | **Gosha-jin-kigan (n = 62)** | **Oxybutynin (po) (n = 4)** | **Tolterodine (n = 0)** |
| --- | --- | --- | --- | --- | --- | --- | --- | --- | --- | --- | --- |
| Day 90 | 59.5%  (n = 1486) | 57.2%  (n = 2464) | 43.3%  (n = 154) | 39.6%  (n = 21) | 42.3%  (n = 113) | 39.0%  (n = 87) | 50.0%  (n = 7) | 26.2%  (n = 11) | 14.5%  (n = 10) | N/A | N/A |
| Day 180 | 43.9%  (n = 1061) | 40.0%  (n = 1690) | 26.6%  (n = 91) | 28.3%  (n = 15) | 26.2%  (n = 68) | 27.5%  (n = 60) | 28.6%  (n = 4) | 11.9%  (n = 5) | 6.5%  (n = 4) | N/A | N/A |
| Day 365 | 28.4%  (n = 687) | 23.6%  (n = 997) | 16.1%  (n = 55) | 15.1%  (n = 8) | 14.2%  (n = 37) | 13.8%  (n = 30) | 7.1%  (n = 1) | 4.8%  (n = 2) | 3.2%  (n = 2) | N/A | N/A |

Supplementary Table S9. Treatment duration of initial OAB medications (median [IQR]) by individual drug, medical specialty, and facility type. Treatment duration was defined as the number of days from the index date to medication change or treatment discontinuation, where discontinuation was defined as no subsequent prescription within 30 days after the end of the previous prescription. Patients who did not experience medication change or treatment discontinuation during follow-up were censored at 365 days. Values are shown as median (IQR). N indicates the number of patients at baseline. N/A indicates not estimable due to no patients. Abbreviations: OAB, overactive bladder; td, transdermal; po, per os.

| **Overall** | **Vibegron** | **Mirabegron** | **Solifenacin** | **Propiverine** | **Fesoterodine** | **Imidafenacin** | **Tolterodine** | **Oxybutynin (td)** | **Oxybutynin (po)** | **Gosha-jin-kigan** | **Flavoxate** |
| --- | --- | --- | --- | --- | --- | --- | --- | --- | --- | --- | --- |
|  | **(n = 22,209)** | **(n = 24,709)** | **(n = 7712)** | **(n = 2065)** | **(n = 2278)** | **(n = 3733)** | **(n = 130)** | **(n = 317)** | **(n = 153)** | **(n = 458)** | **(n = 547)** |
| Median (IQR) | 93.0 (36.0 - 352.0) | 93.0  (37.0 - 324.0) | 84.0 (33.0 - 315.0) | 62.0  (28.0 - 247.0) | 60.0  (28.0 - 182.8) | 60.0  (28.0 - 193.0) | 52.0  (18.5 - 109.5) | 44.0 (21.0 - 109.0) | 37.0 (14.0 - 138.0) | 23.0 (11.0 - 58.0) | 14.0 (7.0 - 40.5) |
|  |  |  |  |  |  |  |  |  |  |  |  |
| **Internal medicine, Clinics** | **Vibegron** | **Mirabegron** | **Solifenacin** | **Fesoterodine** | **Imidafenacin** | **Oxybutynin (td)** | **Oxybutynin (po)** | **Propiverine** | **Tolterodine** | **Gosha-jin-kigan** | **Flavoxate** |
|  | **(n = 8352)** | **(n = 8272)** | **(n = 3742)** | **(n = 1050)** | **(n = 1565)** | **(n = 131)** | **(n = 76)** | **(n = 902)** | **(n = 53)** | **(n = 134)** | **(n = 145)** |
| Median (IQR) | 106.0 (41.0 - 365.0) | 104.0 (41.0 - 365.0) | 91.0 (37.0 - 365.0) | 60.0 (30.0 - 194.0) | 63.0 (30.0 - 255.0) | 55.0 (28.0 - 121.0) | 44.5 (23.2 - 201.0) | 71.0 (30.0 - 316.8) | 60.0 (29.0 - 121.0) | 22.0 (12.0 - 58.0) | 20.0 (7.0 - 53.0) |
|  |  |  |  |  |  |  |  |  |  |  |  |
| **Internal medicine, Hospitals** | **Vibegron** | **Mirabegron** | **Solifenacin** | **Imidafenacin** | **Oxybutynin (po)** | **Fesoterodine** | **Propiverine** | **Oxybutynin (td)** | **Tolterodine** | **Flavoxate** | **Gosha-jin-kigan** |
|  | **(n = 1525)** | **(n = 2563)** | **(n = 951)** | **(n = 215)** | **(n = 12)** | **(n = 195)** | **(n = 230)** | **(n = 23)** | **(n = 12)** | **(n = 36)** | **(n = 25)** |
| Median (IQR) | 133.0  (44.0 - 365.0) | 119.0  (44.0 - 365.0) | 112.0 (43.0 - 365.0) | 105.0 (35.0 - 365.0) | 97.5 (19.8 - 365.0) | 86.0 (36.0 - 253.5) | 81.5 (34.2 - 245.0) | 57.0 (24.5 - 223.0) | 45.5 (26.8 - 102.5) | 29.5 (11.5 - 59.2) | 25.0 (14.0 - 44.0) |
|  |  |  |  |  |  |  |  |  |  |  |  |
| **Urology, Clinics** | **Vibegron** | **Mirabegron** | **Solifenacin** | **Oxybutynin (td)** | **Imidafenacin** | **Fesoterodine** | **Propiverine** | **Tolterodine** | **Gosha-jin-kigan** | **Oxybutynin (po)** | **Flavoxate** |
|  | **(n = 6762)** | **(n = 5317)** | **(n = 718)** | **(n = 49)** | **(n = 984)** | **(n = 356)** | **(n = 297)** | **(n = 22)** | **(n = 150)** | **(n = 15)** | **(n = 241)** |
| Median (IQR) | 70.0 (28.0 - 214.0) | 65.0 (28.0 - 178.0) | 51.5 (17.2 - 149.2) | 47.0 (21.0 - 79.0) | 44.0 (15.0 - 112.2) | 44.0 (14.0 - 107.5) | 39.0 (14.0 - 105.0) | 31.0 (14.0 - 56.2) | 24.5 (11.2 - 57.5) | 22.0 (7.5 - 59.0) | 14.0 (7.0 - 28.0) |
|  |  |  |  |  |  |  |  |  |  |  |  |
| **Urology, Hospitals** | **Vibegron** | **Mirabegron** | **Oxybutynin (td)** | **Solifenacin** | **Imidafenacin** | **Fesoterodine** | **Oxybutynin (po)** | **Propiverine** | **Flavoxate** | **Gosha-jin-kigan** | **Tolterodine** |
|  | **(n = 2416)** | **(n = 4210)** | **(n = 14)** | **(n = 342)** | **(n = 260)** | **(n = 218)** | **(n = 4)** | **(n = 53)** | **(n = 42)** | **(n = 62)** | **(n = 0)** |
| Median (IQR) | 133.0 (51.0 - 365.0) | 118.0 (47.2 - 347.8) | 85.0 (40.8 - 202.8) | 79.0 (30.0 - 210.0) | 72.0 (30.0 - 206.0) | 62.5 (30.0 - 212.5) | 49.0 (33.2 - 67.2) | 46.0 (21.0 - 225.0) | 33.0 (15.0 - 89.8) | 21.0 (9.2 - 56.2) | N/A |
